# Cell surface localization of receptors considered nonfunctional and orphan bitter taste receptors

**DOI:** 10.1016/j.crfs.2026.101551

**Published:** 2026-09-01

**Authors:** Praveen Kumar, Maik Behrens

**Affiliations:** Leibniz Institute for Food Systems Biology at the Technical University of Munich, Lise-Meitner-Str. 34, Freising, 85354, Germany

**Keywords:** TAS2R, GPCR, Heterologous expression assay, Cell surface, Bitter taste, HiBiT

## Abstract

Despite considerable efforts to identify bitter agonists for the ∼25 putatively functional human bitter taste receptors, four receptors remained orphan until now. Whether the problem of identifying agonists for those receptors is due to technical issues related to functional heterologous expression or to the lack of suitable activators is unknown. A similar problem occurs with some variants of bitter taste receptors considered nonfunctional. While some researchers believe that these variants are nonfunctional, others speculate that agonist specificity may have shifted toward yet unknown bitter compounds. Efficient cell-surface localization is generally required for canonical TAS2R signaling in heterologous functional assays and is an important determinant of receptor responsiveness. To address the lack of responsiveness in heterologous expression assays, we quantitatively assessed cell-surface localization of these receptors in living mammalian cells using the HiBiT protein tagging system established in our laboratory. HiBiT-tagged putatively nonfunctional and orphan receptors were expressed in HEK 293T-Gα16gust44 cells and subjected to luminescence measurements using a membrane-impermeable detection reagent. It was observed that all four orphan receptors, TAS2R19, -R42, -R45, and -R60, as well as the putatively nonfunctional receptor variants of TAS2R9, and -R38, were present at the cell surface, albeit at grossly different levels. Immunofluorescence experiments confirmed these results. We conclude that some orphan bitter taste receptors do not show obvious signs of routing or misfolding problems in heterologous cells, suggesting the existence of yet-to-be-discovered bitter activators, particularly for the two receptors, TAS2R19 and TAS2R60, which exhibit pronounced cell-surface localization.

## Highlights

• Limitations of optical cell-surface co-localization experiments were overcome.
• Putative nonfunctional and orphan human TAS2Rs quantitatively assessed on living mammalian cells.
• Orphan TAS2R19 and TAS2R60 are well expressed at the cell surface and may respond to appropriate agonists.
• Diminutive cell surface localization of orphan TAS2R42 and TAS2R45 questions their functionality.

## Graphical abstract

**Figure:**
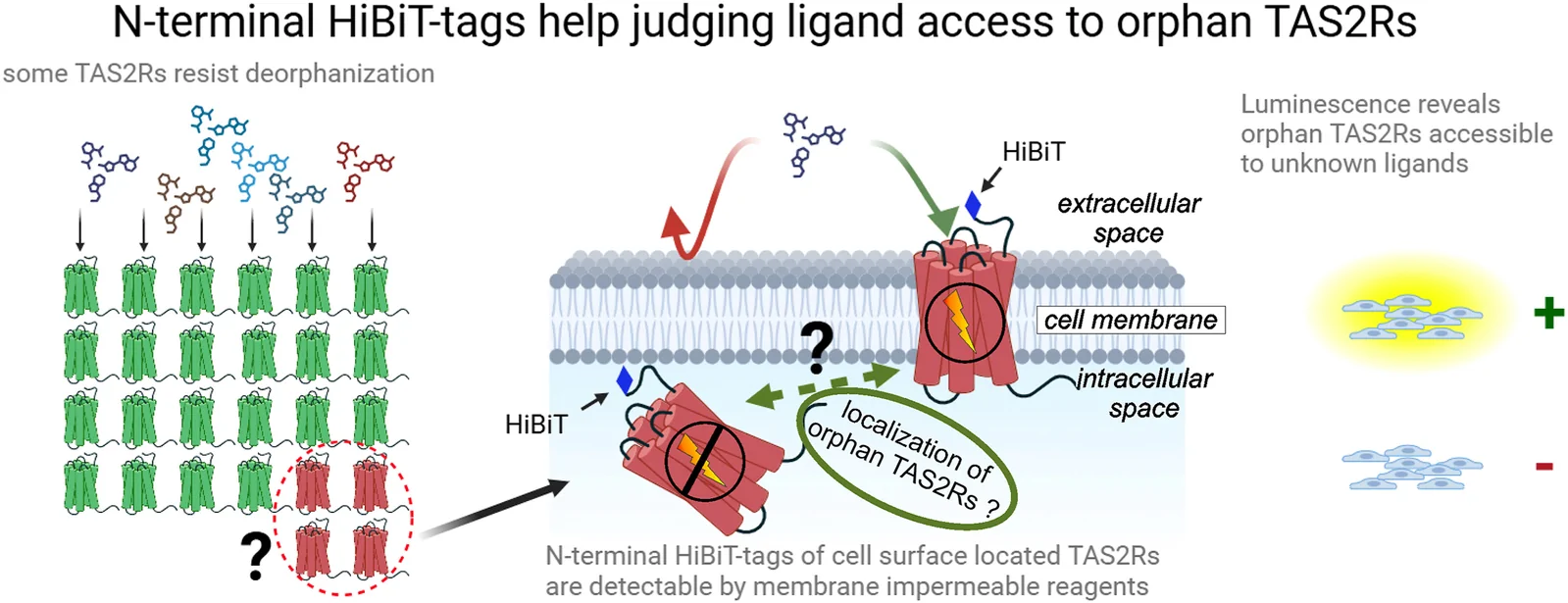

## 1 Introduction

Bitterness perception is a critical biological mechanism that enables humans to identify and avoid potentially toxic substances in foods before ingestion, thereby contributing to survival and health (Drewnowski and Gomez-Carneros, 2000). However, as not all bitter compounds are toxic and sometimes even considered healthy, moderate bitterness is tolerated or even enjoyed by adults (Morini, 2024). The detection of bitter taste is mediated by a family of about 25 bitter taste receptors, which can recognize hundreds of chemically diverse bitter substances (Behrens, 2025; Behrens and Schaefer, 2025; Ziaikin et al., 2025). The human bitter taste receptor family, called taste 2 receptors (TAS2Rs), belongs to the superfamily of G protein-coupled receptors and plays roles in both taste perception and extra-oral physiological processes (Behrens, 2025; Behrens and Schaefer, 2025). The activation profiles of TAS2Rs are highly diverse and partially overlapping, enabling the detection of a vast array of bitter compounds from foods and the environment, with individual variation due to genetic differences (Wooding et al., 2021). During the past decades, the efforts of many labs worldwide have contributed to the identification of bitter activators for most of the ∼25 TAS2Rs (Behrens, 2025; Behrens and Schaefer, 2025; Ziaikin et al., 2025). To date, 22 of 26 TAS2Rs considered as functional have been associated with at least one, sometimes even dozens of bitter activators (Ziaikin et al., 2025; Lang et al., 2023; Meyerhof et al., 2010; Thalmann et al., 2013). However, four receptors, TAS2R19, TAS2R42, TAS2R45, and TAS2R60, resisted de-orphanization until now (Behrens, 2025; Behrens and Schaefer, 2025). Also, among those TAS2Rs considered functional, non-functional variants have been detected. While some putatively nonfunctional variants harbor mutations leading to severe peptide sequence losses, e.g., a truncated variant of the receptor TAS2R46 (Kim et al., 2005; Roudnitzky et al., 2015), others exhibit only single or a few non-synonymous mutations in their coding regions. Whether the latter receptors are indeed non-functional or have simply acquired different agonist profiles cannot be deduced from their amino acid sequence. Two such examples are the receptors TAS2R9 (Dotson et al., 2008) and TAS2R38 (Bufe et al., 2005; Kim et al., 2003). The TAS2R9 variant possessing an alanine residue at amino acid position 187 had been demonstrated to be functional, whereas a valine at this position rendered the receptor non-functional (Dotson et al., 2008). The most famous functional variation in TAS2R genes has been described for the receptor TAS2R38 (Wooding, 2011). The two main variants, TAS2R38-PAV and TAS2R38-AVI, are considered functional and non-functional, respectively, leading to perceptual differences for phenylthiocarbamide (PTC), propyl thiouracil (PROP), as well as other natural and synthetic isothiocyanates/thioureas (Bufe et al., 2005; Kim et al., 2003). While for the TAS2R9-187V variant no speculations about cryptic functionality have been published, for the TAS2R38-AVI a response to an unknown chemical in the pulp of the *Antidesma bunius* fruit was proposed (Risso et al., 2018; Wooding, 2018).

Hence, both orphan receptors and non-functional receptor variants suffer from similar problems, as their functional roles remain unclear until screening assays identify novel activators. Since functional heterologous expression assays are laborious, time-consuming, and expensive, a method to address the overall biosynthetic and structural integrity of these receptor variants would be valuable. We therefore employed a method recently established in our lab that allows the detection and quantification of TAS2Rs at the cell surface of living cells by a high-throughput screening assay. For this, we modified the investigated receptor variants by adding HiBiT (HighBiT)-tags to the amino-terminal end of the already included sst3-export tag (the first 45 amino acids of the rat somatostatin receptor subtype-3). A range of commercially available reagents, some of which are membrane-impermeable and thus enable the detection of extracellular HiBiT-tags on living cells, by binding with high affinity to the HiBiT-sequence added to the receptors. Cell-surface localization indicates that the biosynthesis, folding, and routing of the tested TAS2Rs are apparently normal; this method provides a valuable hint about their functionality, even without knowledge of bitter activators. Furthermore, high-throughput measurements using fluorometric imaging plate readers (FLIPR) in luminescence mode can be performed using the practical, bioluminescence-based detection method.

## 2 Material and methods

### *2.1* Chemicals

Ofloxacin and propyl-2-thiouracil (PROP) were ordered from Sigma-Aldrich. HiBiT extracellular detection reagents, HiBiT lytic detection reagents, and anti-HiBiT monoclonal mouse antibody were purchased from Promega.

### *2.2* Cloning

Modification of the HiBiT-tagged TAS2R14-construct (=TAS2R14M1), which was used as a cloning cassette for all newly generated TAS2R-constructs used in this publication, was done as described previously (Kumar et al., 2026a). Briefly, the 11 amino acids long HiBiT sequence was cloned at the amino-terminal end of the sst3-tag, important for cell-surface routing (Ammon et al., 2002; Bufe et al., 2002). The *Eco*RI restriction site between sst3-tag and the in-frame coding region of TAS2R14 and the *Not*I restriction site between the coding region of TAS2R14 and the in-frame HSV-tag for immunodetection, were cut to replace the TAS2R14 cDNA by the taster variant of TAS2R9 (187Ala) (=TAS2R9M), the non-taster variant of TAS2R9 (187Val) (=TAS2R9NTM), the taster variant of TAS2R38 (PAV) (=TAS2R38M), the non-taster variant of TAS2R38 (AVI) (=TAS2R38NTM), and the orphan receptors TAS2R19 (=TAS2R19M), TAS2R42 (=TAS2R42M), TAS2R45 (=TAS2R45M), and TAS2R60 (=TAS2R60M). For simplicity, we renamed TAS2R14M1 to TAS2R14M accordingly (amino acid sequences of constructs see Supplementary Table S1).

### 2.3 Functional testing of heterologously expressed TAS2R constructs

For the functional characterization of newly modified TAS2Rs, functional assays were carried out in accordance with previous research (Kumar et al., 2026a; Kumar and Behrens, 2024; Lang et al., 2026). Therefore, the HEK293T-Gα16gust44 cells, a generous gift from Jay Slack (Givaudan Flavor Corp.), were seeded to 25 -30% confluency in poly-D-lysine-coated 96-well plates and transiently transfected with the HiBiT-modified TAS2R cDNA (150 ng/well) constructs using (0.5 μL/well) Lipofectamine 2000 (Thermo Fisher Scientific, Darmstadt, Germany). Cells were grown under standard conditions, which included culture media (DMEM, 10% FCS, 1% penicillin/streptomycin, 1% glutamine) and maintaining conditions (37°C, 5% CO_2_, and 100% air humidity). Transfection of an empty vector (mock) served as a negative control. The transfection medium was changed to fresh culture medium after 5 h. After approximately 24 h of incubation, cells were loaded with Fluo4-AM (Thermo Fisher Scientific, Darmstadt, Germany) for 1 h in the presence of probenecid (2.5 mM, Sigma-Aldrich, Steinheim, Germany) to measure fluorescence. Then, C1 buffer (130 mM NaCl, 5 mM KCl, 2 mM CaCl_2_, 10 mM glucose, 10 mM HEPES; pH7.4), was used to wash the cells twice, and the fluorometric imaging plate reader (FLIPR^Tetra^, Molecular Devices, San Jose, CA, USA) was used to track changes in fluorescence (Excitation wavelength: 470-495 nm; emission wavelength: 515-575 nm). Baseline fluorescence was monitored for 18 s, the peak signal was detected at 18 to 38 s after stimulus application. Cell viability was assessed by administering somatostatin 14 (100 nM, Bachem, Bubendorf, Switzerland) to stimulate endogenous somatostatin receptors at the end of each experiment.

### 2.4 Luminescence-based localization of HiBiT-tagged TAS2Rs on the cell surface

The FLIPR^Tetra^ system allowed us to measure both fluorescence and luminescence assays. To investigate the cell-surface localization of TAS2Rs conjugated to a HiBiT sequence via the sst3-tag, HEK293T-Gα16gust44 cells were seeded and transfected (see 2.3). After being incubated for approximately 24 h at 37°C with 5% CO_2_ and saturated air humidity, the cells were washed twice with C1 buffer prior to luminescence measurements (Emission wavelength: 565-625 nm). Nano-Glo® *HiBiT lytic detection* system was used to measure total proteins, while the Nano-Glo® *HiBiT extracellular detection* system was used to measure exclusively receptors at the cell surface (Kumar et al., 2026a). We also compared the responses of unmodified constructs with those of HiBiT-modified TAS2Rs. Briefly, the LgBiT protein (1:100) and the Nano-Glo® HiBiT extracellular or lytic substrate (1:50) were mixed together using the Nano-Glo® HiBiT extracellular or lytic buffer by inversion at room temperature. The wells received a 1:1 (v/v) addition of the resultant Nano-Glo HiBiT extracellular or lytic detection reagent. The exposure time for luminescence measurements was set to 0.53 s. For luminescence measurements, the software Screenworks 4.2 was set to detect the maximum signal (peak) between ∼24 min and ∼32 min after reagent application. Before, the baseline was monitored for ∼32 min.

### 2.5 Immunocytochemistry

To label HEK 293T-Gα16gust44 cells with fluorescence, the cells were seeded on glass coverslips coated with poly-D-lysine (10 μg/mL) under standard conditions for 24 h (Behrens et al., 2019). The following day, plasmids containing TAS2Rs fused with HSV-tags at the C-terminus were transfected into cells using lipofectamine 2000. Cells were incubated on ice for 30 min after being washed twice with pre-warmed (37°C) PBS on the third day. Plasma membranes were stained with biotin-conjugated concanavalin A (1:2000) on ice for 1 h, washed five times with cold PBS, then fixed with methanol-acetone (1:1,v/v) for 2 min on ice. Following multiple PBS washes, unspecific binding was prevented by incubating for 45 min in a solution that contained 0.3% Triton X-100 in PBS and 5% normal horse serum. A mouse anti-HSV antibody (1:15000) was then added for 1 h after being diluted in a modified blocking buffer that contained 0.2% Triton-X-100 in PBS and 5% normal horse serum. The cells were washed four times (5 min each) with PBS before being incubated for 1 h with streptavidin Alexa 633 (1:1000, diluted in modified blocking buffer) and anti-mouse Alexa Fluor 488 (1:2000, diluted in modified blocking buffer) to detect concanavalin A and HSV epitopes, respectively. Nuclei were stained with DAPI (4′,6′-Diamidino-2-Phenylindole; 1 μg/mL) for 15 min in the dark following further PBS rinses. Following three (5 min each) washes with PBS and one with deionized water, cells were embedded in Dako mounting medium. Confocal laser scanning microscopy was used to obtain and analyze immunofluorescence images with a Zeiss LSM 780 device (Carl Zeiss AG, Munich, Germany).

For immunocytochemical live-cell staining of HiBiT-tagged TAS2Rs, a specific antiserum raised against the HiBiT epitope was used as published recently (Kumar et al., 2026a). The cells were cultured and transfected with TAS2R9M, TAS2R9NTM, TAS2R38M, TAS2R38NTM, TAS2R19M, TAS2R42M, TAS2R45M, and TAS2R60M as described (see 2.5). TAS2R60 lacking the HiBiT-tag was used as a negative control. In brief, following PBS washing, cells were placed on ice to suppress endocytosis, and the anti-HiBiT monoclonal antibody (1:1000, in 5% normal horse serum, 1× PBS) was applied for 1 h. The cells were then washed 3 times and fixed (as described above). The cells were again washed 4 times and then incubated with anti-mouse Alexa Fluor 488 (1:2000, diluted in modified blocking buffer) as a secondary antibody. Nuclei were stained with DAPI, and the remaining processes were the same as described in 2.5.

### 2.6 Recording and calculations

For initial testing of compound-receptor pairs, an effective concentration of bitter agonists was applied to cells expressing the individual receptors. The concentration was selected based on previous experiments, taking into account both substance solubility and receptor dependence of compound-induced fluorescence changes (Meyerhof et al., 2010). The same C1 buffer solution was used to dilute the bitter compounds, and at least duplicate wells were made for every compound concentration-receptor combination. To observe the functional response of modified TAS2Rs, cells were seeded, transfected, and stimulated as described for the functional assay procedure (Kumar et al., 2026a). At least three separate experiments provided data for the collection. The data means are displayed along with the standard error of the mean (SEM). We compared the mean peak values of the luminescence assay to the baseline. A value of p ≤ 0.05 was deemed statistically significant using one-way ANOVA and suitable post hoc tests.

## 3 Results

### 3.1 Fusion of the HiBiT sequence to non-functional TAS2R variants allows the study of their cell surface localization

To explore the cell-surface localization of non-functional TAS2R variants, they were subcloned into a HiBiT sequence-containing vector, and fluorescence and luminescence experiments were performed. To test if the addition of the HiBiT sequence affects the functionality of the taster variants of TAS2R9 (Dotson et al., 2008) and TAS2R38 (Bufe et al., 2005), we performed functional assays with TAS2R38-PAV (functional variant without HiBiT-sequence), TAS2R38M (functional variant with HiBiT-sequence), TAS2R38-AVI (putatively nonfunctional variant without HiBiT-sequence), TAS2R38NTM (putatively nonfunctional variant with HiBiT-sequence), TAS2R9-187A (functional variant without HiBiT-sequence), TAS2R9M (functional variant with HiBiT-sequence), TAS2R9-187V (putatively nonfunctional variant without HiBiT-sequence) and TAS2R9NTM (putatively nonfunctional variant with HiBiT-sequence). In the functional assays, we measured the relative fluorescence changes (ΔF/F) of all TAS2R38 and TAS2R9 variants in the presence of PROP (TAS2R38, 100 μM) and ofloxacin (TAS2R9, 2 mM), respectively. We observed activation of TAS2R38-PAV and TAS2R38M in the presence of 100 μM PROP, but not of the non-taster variants TAS2R38-AVI and TAS2R38NTM. Notably, TAS2R38M exhibited a slightly lower response than unmodified TAS2R38-PAV in the fluorescence assay (Fig. 2A), which is consistent with our previously published results (Kumar et al., 2026a). Therefore, HiBiT modification may slightly alter signaling efficiency for individual receptors (e.g., TAS2R38, but not TAS2R14, TAS2R16, and TAS2R46 (Kumar et al., 2026a)) and may not in all cases fully reproduce the behavior of the unmodified constructs. To test TAS2R9 functionality, we used 2 mM ofloxacin and observed similar TAS2R9-187A and TAS2R9M activities in the fluorescence assay. As anticipated, no responses were obtained with the non-taster variants TAS2R9-187V and TAS2R9NTM (Fig. 2B).

**Fig. 1:**
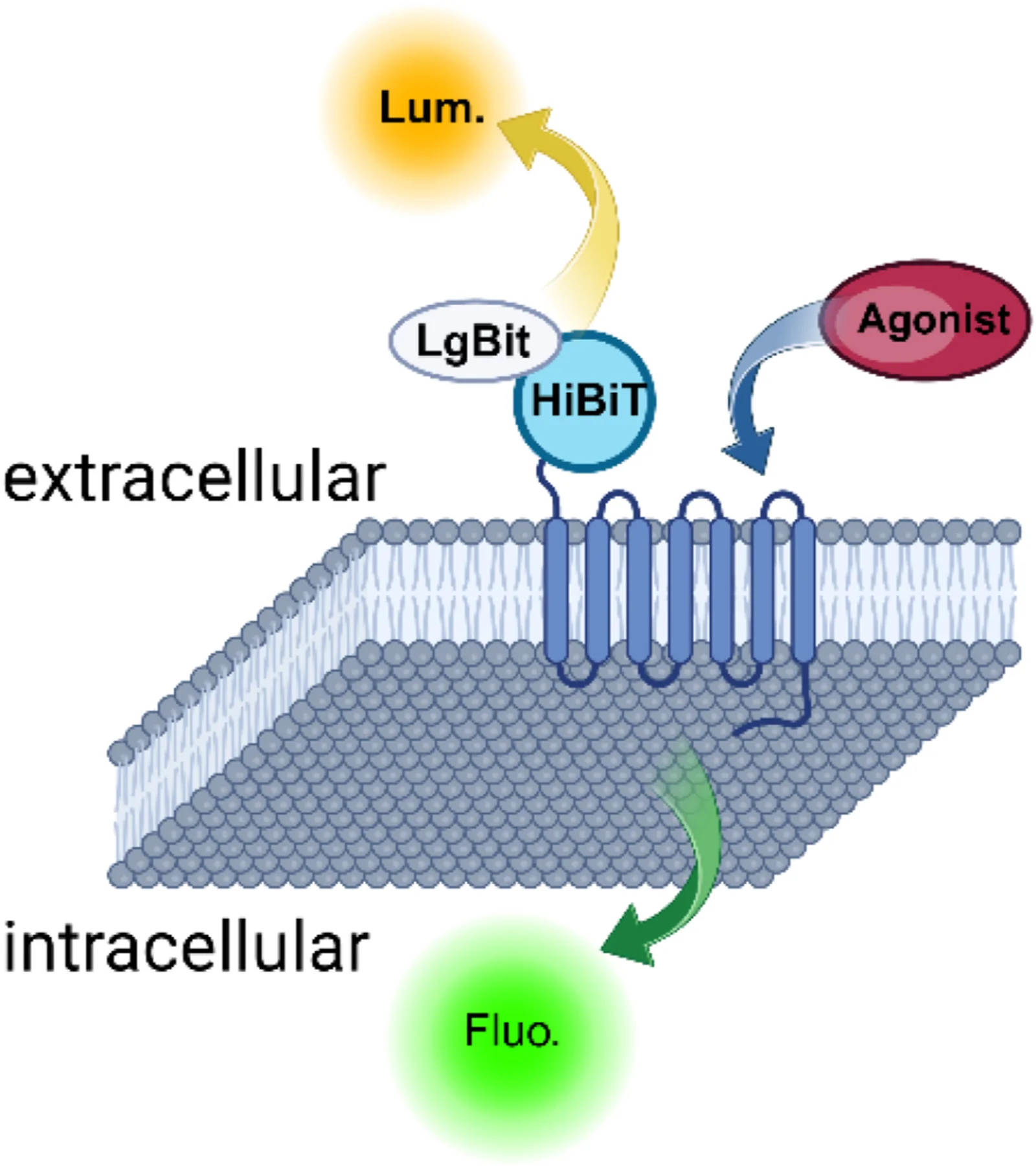
Schematic of assays utilizing the HiBiT/LgBiT reporter for monitoring cell surface localization of TAS2Rs. After agonist stimulation, the GPCR (G protein-coupled receptor) becomes activated, which subsequently activates the intracellular signaling cascade, leading to the release of intracellular calcium and fluorescence detection. The split-luciferase signal, reflecting cell surface-associated HiBiT-tagged protein, is emitted when the luminescence substrate containing an LgBiT sequence binding to HiBiT is added to the cells. Created in BioRender. Behrens, M. (2026) https://BioRender.com/mp9makw.

**Fig. 2:**
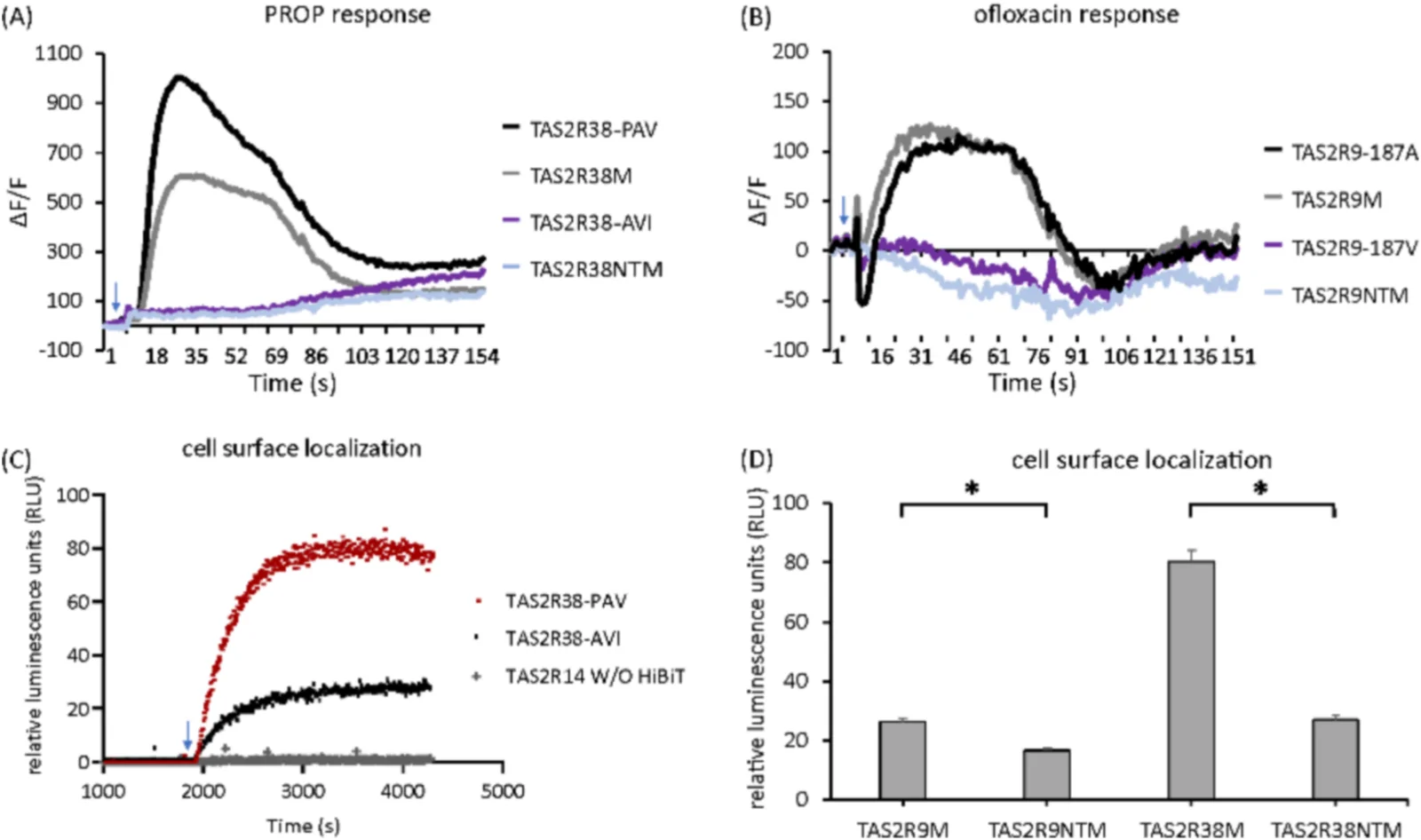
Characterization of TAS2R38-PAV, TAS2R38-AVI, TAS2R9-187A, TAS2R9-187V and their HiBiT-modified variants (NTM and M). TAS2Rs were transiently transfected in HEK 293T-Gα16gust44 cells and subjected to calcium imaging as well as luminescence measurements. (A) Application of PROP (100 μM) to TAS2R38-PAV and TAS2R38-AVI without modification in the sst3-tag and HiBiT-modified variants (TAS2R38M and TAS2R38NTM) transfected HEK 293T-Gα16gust44 cells. (B) Application of ofloxacin (2 mM) to TAS2R9-187A, TAS2R9-187V without modification in the sst3-tag and HiBiT-modified variants (TAS2R9M and TAS2R9NTM) transfected HEK 293T-Gα16gust44 cells. (C) Representative luminescence signal trace of HiBiT-modified TAS2R38M and TAS2R38NTM in the presence of HiBiT extracellular detection reagent. TAS2R14 without HiBiT modification was used as a negative control. (D) Cell surface localization of TAS2R9M and TAS2R9NTM, TAS2R38M and TAS2R38NTM. The y-axis shows the relative fluorescence changes (A + B, ΔF/F) as well as the relative luminescence units (C + D, RLU), and the x-axis (A-C) is labeled with different time scales in the presence of agonist as well as HiBiT extracellular detection reagent. Arrows in panels A-C indicate the time points of stimumlus/reagent applications. Data were collected from 4 (panel 2A), 5 (panel 2B), and 3 (panels 2C and 2D) independent experiments performed in duplicate wells (cf. Supplementary Table S4). Statistical significance was assessed by one-way ANOVA and p < 0.05 was considered significant and indicated by asterisks (for details on statistical analyses see Supplementary Table S2). Data represent means ± standard error of mean (SEM).

To determine the cell surface localization of the HiBiT-tagged TAS2Rs, we performed luminescence assays (Kumar et al., 2026a). The functional TAS2R9M, the putatively nonfunctional TAS2R9NTM, functional TAS2R38M, and the putatively nonfunctional TAS2R38NTM were assayed in the presence of HiBiT extracellular detection reagent for relative luminescence changes (RLU). As a negative control, TAS2R14 without the HiBiT sequence in the sst3-tag was used. We observed noticeable luminescence signals compared to baseline (TAS2R14) with all HiBiT-containing TAS2Rs (Fig. 2C and D). TAS2R38M has a stronger luminescence signal than TAS2R38NTM, indicating that the taster variant of this receptor is more abundant at the cell surface. Similarly, the non-taster variant of TAS2R9 (TAS2R9NTM) is slightly less abundant at the cell surface compared to the taster variant, TAS2R9M (for detailed statistical analyses of luminometric measurements see Supplementary Table S2).

### 3.2 Cell surface localization of HiBiT-tagged orphan bitter taste receptors

**Fig. 3:**
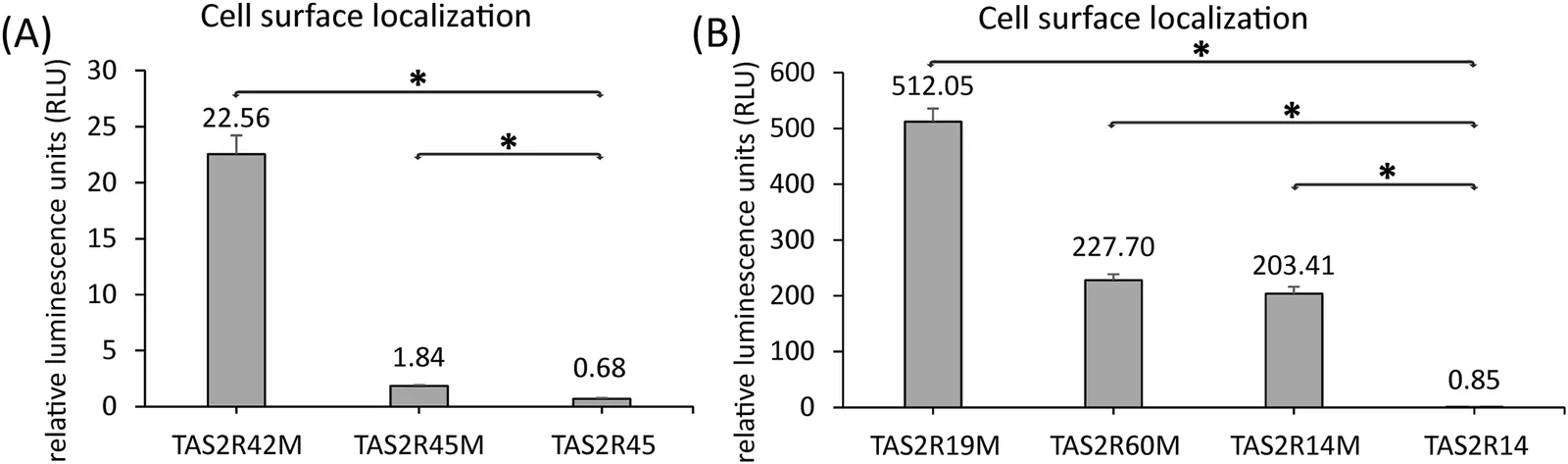
Characterization of cell surface localization of orphan HiBiT-tagged TAS2Rs. TAS2Rs were transiently transfected in HEK 293T-Gα16gust44 cells and subjected to HiBiT extracellular detection. A) Cell surface localization of TAS2R42M, TAS2R45M, and HiBiT-free TAS2R45 (=negative control). B) Cell surface localization of TAS2R19M, TAS2R60M, TAS2R14M (=positive control), and HiBiT-free TAS2R14 (=negative control). The y-axes show (differently scaled in A and B) the relative luminescence units (RLU), and the x-axis is labeled with the different bitter taste receptor constructs. Data were collected from three independent experiments performed in duplicates or triplicates (cf. Supplementary Table S4). Statistical significance was assessed by one-way ANOVA and p < 0.05 was considered significant and indicated by asterisks (for details on statistical analyses see Supplementary Table S2). Data represent means ± standard error of mean (SEM).

To evaluate the cell surface localization of HiBiT-tagged orphan TAS2Rs, luminescence assays were conducted with TAS2R19M, TAS2R42M, TAS2R45M, and TAS2R60M, and relative luminescence changes were measured (RLU). The previously well-studied TAS2R14M with a HiBiT tag was used as a positive control, and a corresponding TAS2R14 lacking a HiBiT tag was used as a negative control. With all HiBiT-tagged TAS2R constructs, notable increases in luminescence signals compared to baseline were observed (Fig. 3A and B). Compared to other HiBiT-containing TAS2Rs, TAS2R19M exhibits a stronger luminescence signal, suggesting that this receptor is more prevalent at the cell surface (Fig. 3B). In contrast, TAS2R45M exhibits only a small luminescence signal (Fig. 3A), suggesting a poor cell-surface localization.

### 3.3 The exact proportion of TAS2Rs reaching the cell surface

In order to investigate whether the abundance of the various TAS2Rs at the cell surface is related to a high amount being produced by the cells, comparative experiments using the HiBiT lytic detection kit in addition were performed to determine the fractions of cell surface-associated TAS2R signals versus the total TAS2R signals.

**Fig. 4:**
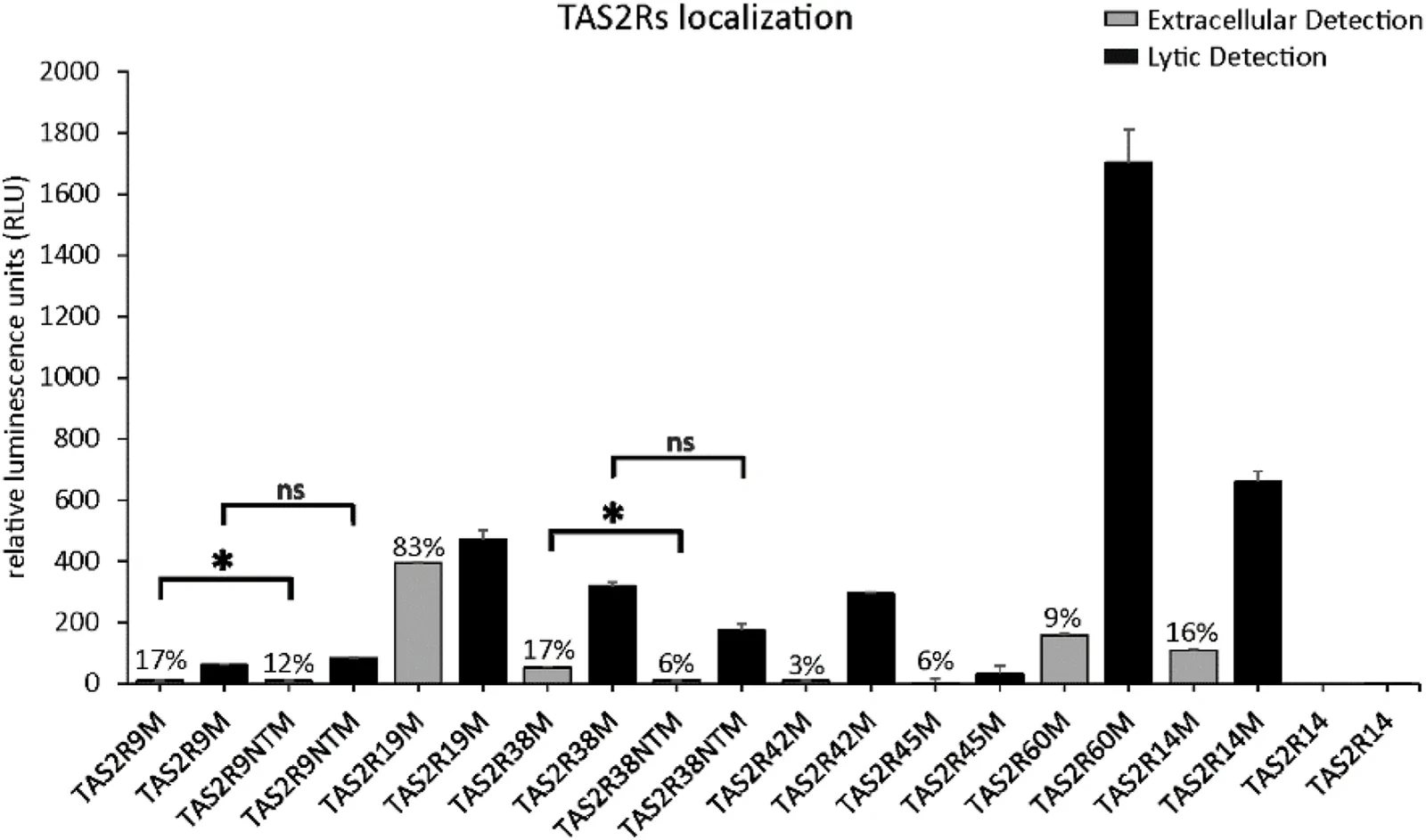
Comparison between extracellular levels vs total levels of HiBiT-tagged TAS2Rs. TAS2Rs were transiently transfected in HEK 293T-Gα16gust44 cells and subjected to HiBiT extracellular detection (gray bars) as well as to HiBiT lytic (=total, black bars) detection. The apparent cell surface fractions of extracellular TAS2R9M (17%), TAS2R9NTM (12%), TAS2R38M (17%), TAS2R38NTM (6%), TAS2R42M (3%), TAS2R45M (6%), TAS2R19M (83%), TAS2R60M (9%), and TAS2R14M (16%) were calculated in percentages by comparing with the total receptor population. A receptor lacking a HiBiT tag (TAS2R14) was used as a negative control and showed negligible relative luminescence units (RLUs). The y-axis shows RLUs, and the x-axis shows the different bitter taste receptor constructs. Data were collected from three independent experiments performed in matched wells from the same transfection in triplicates (cf. Supplementary Table S4). Statistical significance was assessed by one-way ANOVA and p < 0.05 was considered significant and indicated by asterisks (ns = not significant; for details on statistical analyses see Supplementary Table S2). Data represent means ± standard error of mean (SEM).

**Fig. 5:**
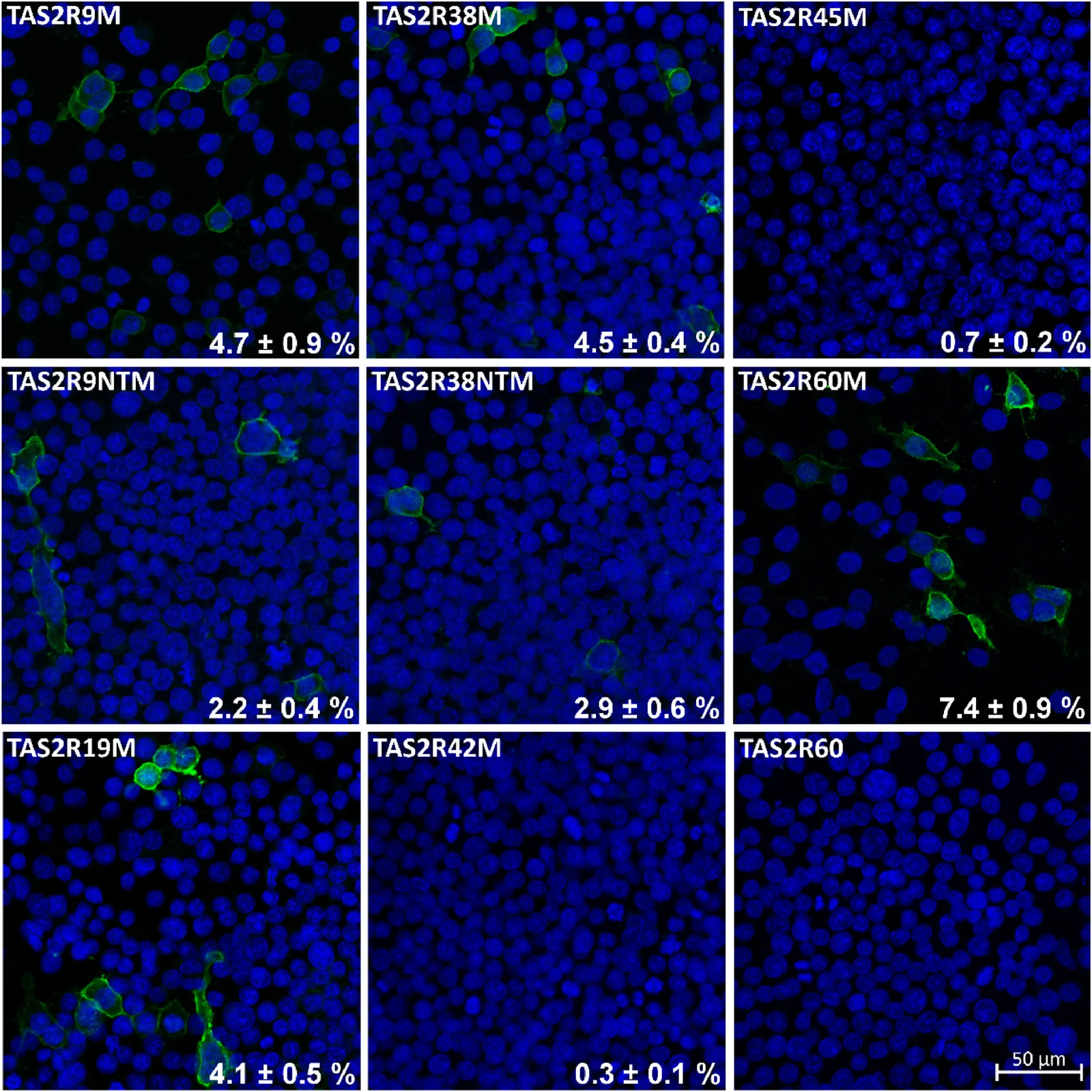
Immunocytochemical staining of TAS2Rs containing HiBiT sequence using an anti-HiBiT antiserum in HEK 293T-Gα16gust44 cells. The TAS2R constructs were transiently transfected into cells, and expression was allowed to continue for ∼24 h. As a negative control, cells were transfected with a TAS2R without the HiBiT sequence (TAS2R60). TAS2Rs on the cell surface were stained in living, unfixed cells with an anti-HiBiT monoclonal antibody. After fixation, cells were incubated with anti-mouse Alexa488 to label HiBiT-tagged TAS2Rs (green), and the nuclei were stained with DAPI (blue). Images were acquired using confocal laser-scanning microscopy (Zeiss LSM 780). Scale bar is provided at the bottom right (TAS2R60). A direct comparison between the HiBiT sequence containing TAS2Rs (TAS2R9M, TAS2R9NTM, TAS2R38M, TAS2R38NTM, TAS2R19M, TAS2R42M, TAS2R45M, TAS2R60M), showing a different pattern of expression. The percentage of receptor expressing cells ± standard error of the mean (one experiment, three fields counted by three assessors) is indicated at the bottom right of the images.

**Fig. 6:**
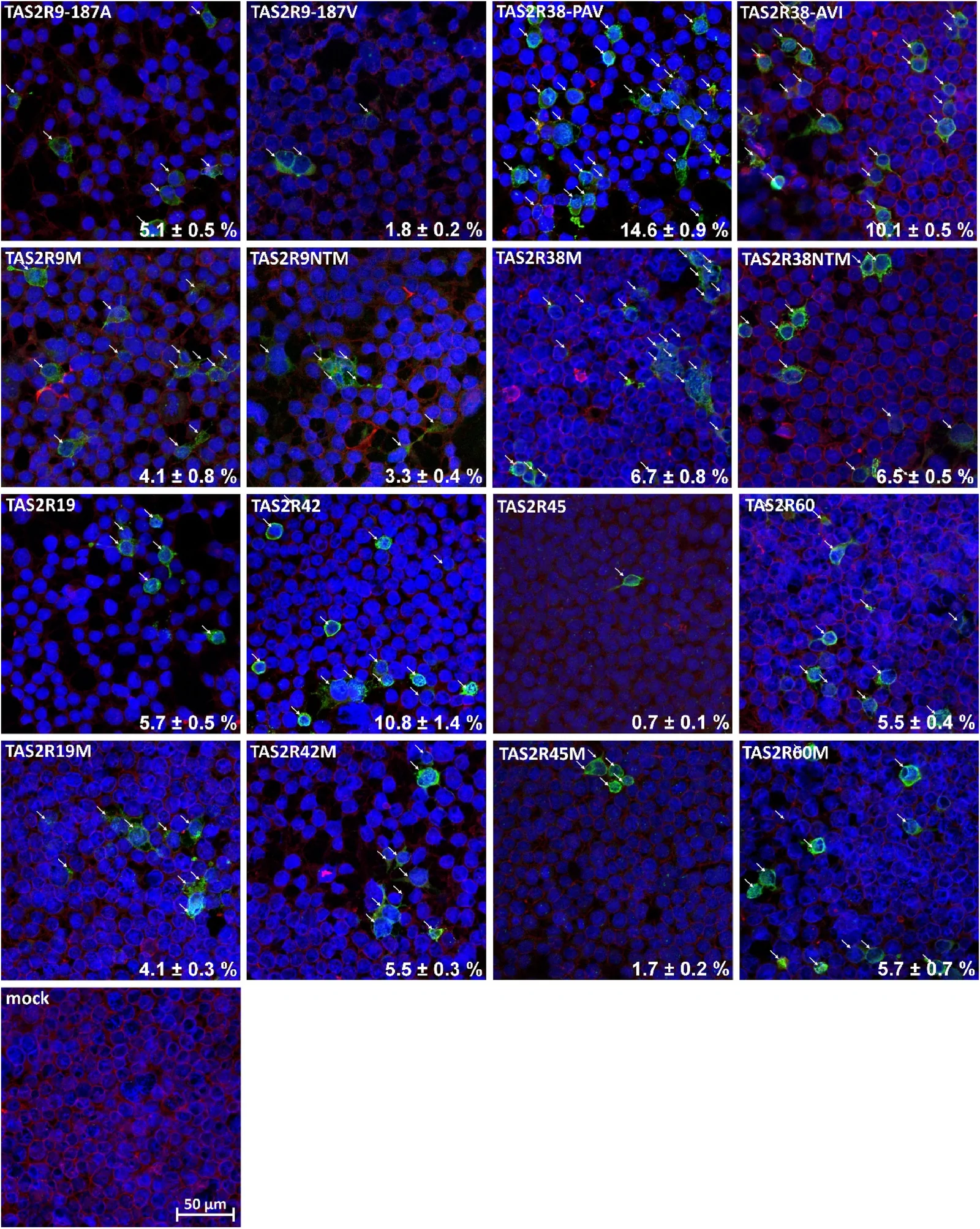
Immunocytochemical staining of TAS2Rs (with and without HiBiT sequence) in HEK 293T-Gα16gust44 cells. To demonstrate the overall expression of TAS2Rs, the TAS2R constructs were transiently transfected into cells, and expression was allowed to continue for ∼24 h. Cells were transfected with an empty expression vector as a negative control (mock). Following fixation, cells were treated with anti-HSV antibodies and anti-mouse Alexa488 to label HSV-tagged TAS2Rs (green), and the cell surface was marked with biotinylated concanavalin A and streptavidin-Alexa633 (red). DAPI was used to stain the nuclei (blue). Confocal laser-scanning microscopy (Zeiss LSM 780) was used to capture the images. The images display channel overlays of green, red, and blue. Overlapping signals from receptor and cell-surface staining are indicated by arrows. Scale bar, bottom left (mock). The cell surface localization of TAS2Rs lacking HiBiT-tags (TAS2R9-187A, TAS2R9-187V, TAS2R38-PAV, TAS2R38-AVI, TAS2R19, TAS2R42, TAS2R45, TAS2R60) and the HiBiT-tagged sst3-TAS2Rs (TAS2R9M, TAS2R9NTM, TAS2R38M, TAS2R38NTM, TAS2R19M, TAS2R42M, TAS2R45M, and TAS2R60M), demonstrating similar expression patterns. The percentage of receptor expressing cells ± standard error of the mean (2 experiments with two glass coverslips for TAS2R9 and TAS2R9NT; all others, 1 experiment with two glass coverslips; three fields counted by three assessors) is indicated.

The results of these experiments demonstrate that the total amounts of the different TAS2Rs fluctuate considerably (Fig. 4). Whereas the production of TAS2R60 exceeded by far the total levels of all other TAS2Rs, and, in particular, that of TAS2R45, the plasma membrane-associated fractions deviated less pronouncedly. The smallest fractions of cell surface-associated receptors were identified for TAS2R42M (3%), TAS2R38NTM (6%), for TAS2R45M (6%), and for TAS2R60M (9%), although the latter receptor was found to be produced at extremely high amounts. Several TAS2Rs, TAS2R9M (17%), TAS2R9NTM (12%), TAS2R38M (17%), and TAS2R14M (16%), exhibited a cell surface-associated fraction of 12% to 17%. With a high level of cell-surface localization (83%), TAS2R19M stood out. Immunocytochemical experiments confirmed the results of the HiBiT lytic and membrane-impermeable detection (Fig. 5, Fig. 6).

### 3.4 Immunocytochemical detection of HiBiT-tagged TAS2Rs

HiBiT-based immunocytochemical staining was performed to assess whether the HiBiT-tag is exposed on the extracellular side of the plasma membrane. We observed very low or no signal for TAS2R42M and TAS2R45M. Only occasionally were single cells with green signals observed. These seemed to be damaged cells and not representative of the overall outcome of the experiment. Therefore, we excluded these cells from the images (cf. Supplementary Fig. S1). The lack of cells stained for extracellular TAS2R42M and TAS2R45M indicates that these receptors are poorly trafficked and possibly mostly retained in the ER. In addition, TAS2R9M, TAS2R9NTM, TAS2R38M, TAS2R38NTM, TAS2R19M, and TAS2R60M are well expressed on the cell surface.

Immunocytochemical staining with anti-HSV antibodies detecting the intracellular carboxy terminus of TAS2Rs was performed to examine the intra- and extracellular localization of TAS2Rs and compare it with that of HiBiT-containing TAS2Rs. We observed that orphan and receptors considered nonfunctional are expressed in cells. In addition, a direct comparison of the localization of functional and putatively nonfunctional variants of TAS2R9 and TAS2R38 reveals similar expression patterns. Our previous findings indicate that the HiBiT sequence does not profoundly affect TAS2R expression levels. Here, we strengthened the evidence by comparing HiBiT- and non-HiBiT-containing TAS2R sequences immunocytochemically.

## 4 Discussion

Since their discovery in the year 2000 (Adler et al., 2000; Chandrashekar et al., 2000; Matsunami et al., 2000), it has been of considerable interest to identify bitter activators for human and other species’ bitter taste receptors. Because of limited substance amount, toxicities, and the need for high-throughput analyses, most of the research was done using functional heterologous expression assays (for a recent review see (Behrens, 2025)). While, due to the efforts of many groups worldwide, the identification of bitter agonists for human TAS2Rs was very successful, leading to the deorphanization of 22 of 26 TAS2Rs considered functional (Lang et al., 2023; Meyerhof et al., 2010; Thalmann et al., 2013), for four receptors, no agonists have been found so far. In general, there exist two possible reasons for the lack of cognate agonists for the four orphan TAS2Rs: a) technical issues arising from the functional expression of these chemoreceptors, which are considered to be difficult to express heterologously (Bufe et al., 2002; Chandrashekar et al., 2000; Behrens et al., 2006), or b) a lack of the appropriate bitter compounds for their activation. While the latter reason is rather trivial and may require the isolation of bitter compounds from so far neglected sources (see (Schmitz et al., 2025) and discussion therein), technical issues could be manifold. One possible technical issue could be a lack of coupling to the signaling cascade present in the employed cell lines, although dozens of bitter taste receptors of species ranging from human (Meyerhof et al., 2010) to, most recently, cartilaginous fish (Behrens et al., 2023), have been successfully subjected to screening in the same cell system.

The existence of numerous cell lines endogenously expressing human (e.g., HuTu-80, NCI-H716 (Mori et al., 2022)) or mouse (e.g., STC-1 (Wu et al., 2002)) bitter taste receptors may represent another approach to overcome potential technical issues with HEK 293 cells. However, many of these cell lines are immortalized or cancer cell lines, which may not provide the desired “more natural” cellular environment suitable for deorphanization of bitter taste receptors. Moreover, they typically express numerous receptors (Mori et al., 2022; Wu et al., 2002), making it difficult to assess the function of individual receptors. Unfortunately, there exists a persistent lack of commercially available, specific, and validated antisera for bitter taste receptors (Behrens and Schaefer, 2025; Behrens et al., 2012 ), which often results in RT-PCR data serving as the sole confirmation of expression. As most cell lines are of non-gustatory origin and it has been shown that there is a large difference between gustatory and most non-gustatory expression levels (Prandi et al., 2013; Voigt et al., 2012 ), the questionable biological significance of PCR-detected bitter taste receptor mRNA levels is another issue for employing such cell lines for bitter compound screening. Many, but not all, of these problems might be overcome in the future by establishing tissue-specific organoids (Kumar et al., 2026b), which, combined with genetic engineering, could become improved tools for bitter taste receptor research in more natural environments.

Another possible cause for the inability to identify agonists for the remaining four TAS2Rs by heterologous expression could be related to their biosynthesis, protein stability, and cell surface routing (Behrens et al., 2006). Efficient cell-surface localization is generally required for canonical TAS2R signaling in heterologous functional assays and is an important determinant of receptor responsiveness. This requires successful biosynthesis, sufficient protein stability, as well as proper execution of maturation and trafficking steps. The present work was devoted to analyzing the cell surface localization of orphan TAS2Rs and TAS2R variants considered non-functional.

To address the cell surface localization of TAS2Rs in living cells, a recently established method, the addition of HiBiT-tags at the amino terminus of the sst3-export-tag (Ammon et al., 2002; Bufe et al., 2002) was employed (Kumar et al., 2026a). This method not only allows the use of membrane-impermeable detection reagents, but also the utilization of a high-throughput device, the fluorometric imaging plate reader (FLIPR). Furthermore, the accurate quantification of luminescence signals emitted from a large pool of identically treated cells is possible. Moreover, another benefit is the possibility of directly correlating receptor function with cell surface association (Fig. 1), as has been established recently (Kumar et al., 2026a).

The first experiment of this study was devoted to elucidating the cell surface localization of two non-functional TAS2R variants, the TAS2R9-187V and the TAS2R38-AVI. The non-functional TAS2R9 variation was discovered when investigating genes associated with impaired glucose homeostasis and increased diabetes risk in a rather isolated living population (Dotson et al., 2008). The study identified functional (TAS2R9-187A) and non-functional (TAS2R9-187V) variants of this receptor in the investigated population and showed its expression in a cell model of human enteroendocrine L-cells (NCI-H716) (Jang et al., 2007). Whereas three bitter drugs, ofloxacin, procainamide, and pirenzepine, only activated TAS2R9-187A, the TAS2R9-187V was not activated (Dotson et al., 2008). The localization of position 187 in TAS2R9 in the upper part of transmembrane domain (TM) five (https://gpcrdb.org/protein/ta2r9_human/) does not exclude participation in agonist binding; hence, instead of a loss-of-function for TAS2R9-187V, a change in ligand binding would be conceivable. Our data showing the moderate reduction in cell surface association of TAS2R9-187V compared to TAS2R-187A (Fig. 2, Fig. 4) would suggest that no gross changes in folding and routing preclude functionality of this receptor variant. This is confirmed by the immunocytochemical experiment on living, non-permeabilized cells performed with the anti-HiBiT antiserum (Fig. 5). Therefore, the functionality of this receptor challenged with a different and perhaps smaller agonist appears possible. Of course, the bulkiness of valine compared to alanine could sterically affect the binding of ligands into the binding pocket of TAS2R9 or limit the necessary movements of TMs required for the conformational changes to adopt the active state conformation in general.

The data obtained for the functional and non-functional variants of TAS2R38 suggest a similar scenario for this receptor, although the ratio of non-functional TAS2R38-AVI at the cell surface is reduced compared to TAS2R38-PAV (Fig. 4). Moreover, the total luminescence observed in Fig. 2 is visibly reduced compared to TAS2R38-PAV. In contrast, the cell surface association and overall expression assessed by immunocytochemical staining experiments (Fig. 5, Fig. 6) did not reveal obvious differences. The two main variants of TAS2R38 account for the pronounced variance of bitter PROP/PTC-tasting in the human population (Bufe et al., 2005; Kim et al., 2003), although smaller contributions of other TAS2R variants, e.g., of the receptor TAS2R4, are reported (Nolden et al., 2024). Whereas some studies based on structure-function analyses of TAS2R38 suggested the involvement of polymorphic sites in this receptor on ligand interactions (Tan et al., 2012), others proposed that a general sterical hindrance is caused in particular by a pair of residues, including position 296, the V/I in PAV or AVI (Biarnés et al., 2010). In fact, for the apparent non-functional TAS2R38-AVI, it was suggested that a change in ligand specificity occurred, from isothiocyanates/thioureas to yet unknown compound(s) present in the fruit *Antidesma bunius* (Risso et al., 2018; Wooding, 2018). Unless the relevant *Antidesma* compounds were isolated and tested for TAS2R38-AVI activation, it will remain an open question whether TAS2R38-AVI is, in principle, functional or not. Nevertheless, the data presented in this report underscores the presence of TAS2R38-AVI at the cell surface, indicating an apparent structural integrity/accurate routing of this variant.

In the course of the experiments performed in the current study, all four orphan receptors, TAS2R19, TAS2R42, TAS2R45, and TAS2R60, could be detected by luminescence experiments at the cell surface of HEK 293T-Gα16gust44 cells (Fig. 3, Fig. 4, Fig. 5), albeit TAS2R42 and TAS2R45 showed only negligible immunofluorescent signals when stained with the anti-HiBiT antiserum (Fig. 5). In contrast to TAS2R45, which seems to be synthesized only at a very low level (Fig. 4) and shows only a small amount of receptor at the cell surface (Fig. 3, Fig. 4), TAS2R42 is synthesized quite well, but not routed very effectively to the cell surface. The other two TAS2Rs are readily produced and reach the cell surface. Therefore, it appears reasonable to assume that biosynthesis and routing of TAS2R19 and TAS2R60 in heterologous cells do not account for their orphan state, in contrast to TAS2R42 and TAS2R45, which could resist de-orphanization and might even represent expressed pseudogenes. In particular, the TAS2R19 stands out because it is even more abundant at the cell surface than TAS2R14, which has, by far, the most cognate agonists of all human TAS2Rs. The TAS2R60 is by far the strongest expressed receptor subtype, and even though the fraction of TAS2R60 reaching the cell surface is lower than that of other receptors, the total amount at the cell surface exceeds all TAS2Rs except TAS2R19.

Another possible cause for the, at present, non-functionality of TAS2R19 and TAS2R60 could be a lack of coupling of these receptors to the calcium signaling cascade. This appears unlikely since both receptors are not remotely related members of the bitter taste receptor gene family, they are closely related to already de-orphaned members of e.g., mouse Tas2rs: The mouse receptor Tas2r135 is an ortholog of TAS2R60 and has currently 11 identified bitter agonists (Lossow et al., 2016), the TAS2R19 is highly homologous to the TAS2R20 with currently 10 known agonists (Ziaikin et al., 2025), this is in sharp contrast to TAS2R42 which is related to mouse Tas2r131, another receptor which resisted de-orphanization until now (Lossow et al., 2016). In light of the many, much more remotely related bitter taste receptors identified in the same cellular system until now, problems with the coupling to the signaling cascade seem unlikely to be the cause for the lack of functionality.

In this engineered heterologous expression system, gross cell-surface trafficking failure is unlikely to be the sole explanation for the orphan status of TAS2R19, and possibly TAS2R60. These receptors may therefore be reasonable candidates for further ligand screening, but their signaling competence remains to be demonstrated. In fact, our current knowledge about our bitter surroundings is patchy. Whereas numerous bitter compounds of synthetic origin as well as bitter compounds from flowering plants have been used for functional bitter taste receptor screenings (cf. (Ziaikin et al., 2025)), few compounds from animals, mushrooms/fungi, prokaryotic organisms, or plants from older branches of the plant kingdom have been identified as bitter and used for screening. This serious knowledge gap needs to be closed by intensified research.

## Data availability:

All data from this study are provided in the main text and supplementary information. Further inquiries can be directed to the corresponding author.

## Author contributions

All authors contributed to the study conception and design. Material preparation, data collection and analysis were performed by Praveen Kumar. The first draft of the manuscript was written by Praveen Kumar and all authors commented on previous versions of the manuscript. All authors read and approved the final manuscript.

## Funding sources

The authors declare that no funds, grants, or other support were received during the preparation of this manuscript.

## Declaration of competing interest

The authors declare that they have no known competing financial interests or personal relationships that could have appeared to influence the work reported in this paper.

## References

[bib1] Adler E., Hoon M.A., Mueller K.L., Chandrashekar J., Ryba N.J., Zuker C.S. (2000). A novel family of mammalian taste receptors. Cell.

[bib2] Ammon C., Schäfer J., Kreuzer O.J., Meyerhof W. (2002). Presence of a plasma membrane targeting sequence in the amino-terminal region of the rat somatostatin receptor 3. Arch. Physiol. Biochem..

[bib3] Behrens M. (2025). International union of basic and clinical pharmacology. CXVII: taste 2 receptors-Structures, functions, activators, and blockers. Pharmacol. Rev..

[bib4] Behrens M., Bartelt J., Reichling C., Winnig M., Kuhn C., Meyerhof W. (2006). Members of RTP and REEP gene families influence functional bitter taste receptor expression. J. Biol. Chem..

[bib5] Behrens M., Lang T., Korsching S.I. (2023). A singular shark bitter taste receptor provides insights into the evolution of bitter taste perception. Proc. Natl. Acad. Sci. U. S. A..

[bib6] Behrens M., Redel U., Blank K., Meyerhof W. (2019). The human bitter taste receptor TAS2R7 facilitates the detection of bitter salts. Biochem. Biophys. Res. Commun..

[bib7] Behrens M., Schaefer S. (2025). Bitter taste receptors. Chem. Senses.

[bib8] Behrens Maik, Born Stephan, Redel Ulrike, Voigt Nadine, Schuh Vanessa, Raguse Jan-Dirk, Meyerhof Wolfgang, Glendinning John I. (2012). Immunohistochemical Detection of TAS2R38 Protein in Human Taste Cells. PLoS ONE.

[bib9] Biarnés X., Marchiori A., Giorgetti A., Lanzara C., Gasparini P., Carloni P., Born S., Brockhoff A., Behrens M., Meyerhof W. (2010). Insights into the binding of phenyltiocarbamide (PTC) agonist to its target human TAS2R38 bitter receptor. PLoS One.

[bib10] Bufe B., Breslin P.A., Kuhn C., Reed D.R., Tharp C.D., Slack J.P., Kim U.K., Drayna D., Meyerhof W. (2005). The molecular basis of individual differences in phenylthiocarbamide and propylthiouracil bitterness perception. Curr. Biol..

[bib11] Bufe B., Hofmann T., Krautwurst D., Raguse J.D., Meyerhof W. (2002). The human TAS2R16 receptor mediates bitter taste in response to beta-glucopyranosides. Nat. Genet..

[bib12] Chandrashekar J., Mueller K.L., Hoon M.A., Adler E., Feng L., Guo W., Zuker C.S., Ryba N.J. (2000). T2Rs function as bitter taste receptors. Cell.

[bib13] Dotson C.D., Zhang L., Xu H., Shin Y.K., Vigues S., Ott S.H., Elson A.E., Choi H.J., Shaw H., Egan J.M. (2008). Bitter taste receptors influence glucose homeostasis. PLoS One.

[bib14] Drewnowski A., Gomez-Carneros C. (2000). Bitter taste, phytonutrients, and the consumer: a review. Am. J. Clin. Nutr..

[bib15] Jang H.J., Kokrashvili Z., Theodorakis M.J., Carlson O.D., Kim B.J., Zhou J., Kim H.H., Xu X., Chan S.L., Juhaszova M. (2007). Gut-expressed gustducin and taste receptors regulate secretion of glucagon-like peptide-1. Proc. Natl. Acad. Sci. U. S. A..

[bib16] Kim U., Wooding S., Ricci D., Jorde L.B., Drayna D. (2005). Worldwide haplotype diversity and coding sequence variation at human bitter taste receptor loci. Hum. Mutat..

[bib17] Kim U.K., Jorgenson E., Coon H., Leppert M., Risch N., Drayna D. (2003). Positional cloning of the human quantitative trait locus underlying taste sensitivity to phenylthiocarbamide. Science.

[bib18] Kumar P., Behrens M. (2024). Influence of sodium chloride on human bitter taste receptor responses. J. Agric. Food Chem..

[bib19] Kumar P., Ferri F., Di Pizio A., Behrens M. (2026). Human bitter taste receptors undergo internalization in an agonist-selective fashion. Int. J. Biol. Macromol..

[bib20] Kumar P., Ziegler F., Behrens M. (2026). Establishing murine intestinal organoids to study Nutrient- and Tastant-Evoked gut signaling. Nutrients.

[bib21] Lang T., Di Pizio A., Risso D., Drayna D., Behrens M. (2023). Activation profile of TAS2R2, the 26th human bitter taste receptor. Mol. Nutr. Food Res..

[bib22] Lang T., Ferri F., Ziegler F., Pizio A.D., Behrens M. (2026). Steroid hormones are potent and putatively endogenous activators of human bitter taste receptors. Ann. N. Y. Acad. Sci..

[bib23] Lossow K., Hübner S., Roudnitzky N., Slack J.P., Pollastro F., Behrens M., Meyerhof W. (2016). Comprehensive analysis of mouse bitter taste receptors reveals different molecular receptive ranges for orthologous receptors in mice and humans. J. Biol. Chem..

[bib24] Matsunami H., Montmayeur J.P., Buck L.B. (2000). A family of candidate taste receptors in human and mouse. Nature.

[bib25] Meyerhof W., Batram C., Kuhn C., Brockhoff A., Chudoba E., Bufe B., Appendino G., Behrens M. (2010). The molecular receptive ranges of human TAS2R bitter taste receptors. Chem. Senses.

[bib26] Mori Y., Aoki A., Okamoto Y., Isobe T., Ohkawara S., Hanioka N., Tanaka-Kagawa T., Jinno H. (2022). Isoform-specific quantification of human bitter taste receptor transcripts using real-time PCR analysis. Biol. Pharm. Bull..

[bib27] Morini G. (2024). The taste for health: the role of taste receptors and their ligands in the complex food/health relationship. Front. Nutr..

[bib28] Nolden A.A., Behrens M., McGeary J.E., Meyerhof W., Hayes J.E. (2024). Differential activation of TAS2R4 may recover ability to taste propylthiouracil for some TAS2R38 AVI homozygotes. Nutrients.

[bib29] Prandi Simone, Bromke Marta, Hübner Sandra, Voigt Anja, Boehm Ulrich, Meyerhof Wolfgang, Behrens Maik, Glendinning John I. (2013). A Subset of Mouse Colonic Goblet Cells Expresses the Bitter Taste Receptor Tas2r131. PLoS ONE.

[bib30] Risso D., Sainz E., Morini G., Tofanelli S., Drayna D. (2018). Taste perception of Antidesma bunius fruit and its relationships to bitter taste receptor gene haplotypes. Chem. Senses.

[bib31] Roudnitzky N., Behrens M., Engel A., Kohl S., Thalmann S., Hübner S., Lossow K., Wooding S.P., Meyerhof W. (2015). Receptor polymorphism and genomic structure interact to shape bitter taste perception. PLoS Genet..

[bib32] Schmitz L.M., Lang T., Steuer A., Koppelmann L., Di Pizio A., Arnold N., Behrens M. (2025). Taste-guided isolation of bitter compounds from the mushroom Amaropostia stiptica activates a subset of human bitter taste receptors. J. Agric. Food Chem..

[bib33] Tan, J, Abrol, R, Trzaskowski, B, Goddard, W A, 3rd (2012) 3D structure prediction of TAS2R38 bitter receptors bound to agonists phenylthiocarbamide (PTC) and 6-n-propylthiouracil (PROP). J. Chem. Inf. Model. 52, 1875-1885 10.1021/ci300133a. 22656649

[bib34] Thalmann S., Behrens M., Meyerhof W. (2013). Major haplotypes of the human bitter taste receptor TAS2R41 encode functional receptors for chloramphenicol. Biochem. Biophys. Res. Commun..

[bib35] Voigt A., Hubner S., Lossow K., Hermans-Borgmeyer I., Boehm U., Meyerhof W. (2012). Genetic Labeling of Tas1r1 and Tas2r131 Taste Receptor Cells in Mice. Chemical Senses.

[bib36] Wooding S. (2011). Signatures of natural selection in a primate bitter taste receptor. J. Mol. Evol..

[bib37] Wooding S.P. (2018). Bitter fruit: inverse associations between PTC and Antidesma bunius perception. Chem. Senses.

[bib38] Wooding S.P., Ramirez V.A., Behrens M. (2021). Bitter taste receptors: genes, evolution and health. Evol. Med. Publ. Health.

[bib39] Wu S.V., Rozengurt N., Yang M., Young S.H., Sinnett-Smith J., Rozengurt E. (2002). Expression of bitter taste receptors of the T2R family in the gastrointestinal tract and enteroendocrine STC-1 cells. Proc. Natl. Acad. Sci. U. S. A..

[bib40] Ziaikin E., David M., Uspenskaya S., Niv M.Y. (2025). BitterDB: 2024 update on bitter ligands and taste receptors. Nucleic Acids Res..

