# Supplementary material for: Cell surface localization of receptors considered nonfunctional and orphan bitter taste receptors
Source: Curr Res Food Sci. 2026 Sep 1;13:101551. doi: 10.1016/j.crfs.2026.101551 (PMC13571512; doi:10.1016/j.crfs.2026.101551)
Supplement: Supplementary file 1 [file mmc1.docx]

**Supplementary Information**

**Cell surface localization of nonfunctional and orphan bitter taste receptors**

**Praveen Kumar, and Maik Behrens^*^**

Leibniz Institute for Food Systems Biology at the Technical University of Munich, Lise-Meitner-Str. 34, 85354 Freising, Germany

**Content**

Supplementary Figure S1 (pages 2 – 4)	Detailed immunocytochemical analysis of HiBiT-tagged TAS2R42, TAS2R45, and TAS2R19 surface and total expression in HEK293T-Gα16gust44 cells.

Supplementary Table S1 (page 5)	Amino acid sequences of the used TAS2Rs and their variants.

Supplementary Table S2 (pages 6 – 8)	Detailed statistics for luminometric measurements shown in Figures 2, 3, and 4 of the main manuscript.

Supplementary Table S3 (page 9)	Raw data of cell surface and total luminescence detection.

Supplementary Table S4 (page 9)	Replicate level numerical data for experiments.

**Supplementary Figure S1**

**Immunocytochemical detection of HiBiT-tagged TAS2Rs**

Surface and subcellular localization of HiBiT-tagged bitter taste receptors (TAS2Rs) was assessed by immunocytochemistry (ICC) using a monoclonal antibody directed against the HiBiT epitope . HEK293T-Gα16gust44 cells were seeded and transiently transfected with expression constructs encoding TAS2R9M, TAS2R9NTM, TAS2R38M, TAS2R38NTM, TAS2R19M, TAS2R42M, TAS2R45M, and TAS2R60M as detailed in section 2.5. An untagged TAS2R60 construct served as a negative control for HiBiT-specific staining.

**Live-cell surface staining:** To evaluate plasma membrane expression, cells were washed with phosphate-buffered saline (PBS), cooled on ice to halt endocytosis, and incubated for 1 h with anti-HiBiT monoclonal antibody (1:1000) diluted in PBS containing 5% normal horse serum. After three PBS washes, cells were fixed according to the protocol in section 2.5, washed an additional four times, and incubated with Alexa Fluor 488-conjugated anti-mouse secondary antibody (1:2000) in modified blocking buffer. Nuclei were counterstained with DAPI, and all subsequent steps followed the standard procedure described in section 2.5.

**Combined surface and total receptor visualization:** To distinguish surface-localized versus intracellular pools of TAS2R42M, TAS2R45M, and TAS2R19M, two complementary ICC approaches were employed: **1) Concanavalin A co-staining under live-cell conditions**: Cells were processed as above for surface staining, with the addition of concanavalin A to the primary antibody incubation to label the plasma membrane. **2) Fixed-cell staining for total receptor detection**: Following PBS washing and cooling on ice, cells were first incubated with concanavalin A for 1 h, then fixed. Subsequently, anti-HiBiT antibody (1:1000 in 5% normal horse serum, 1× PBS) was applied for 1 h to permit access to intracellular epitopes. All remaining steps were performed according to the standard ICC protocol in section 2.5.

**Image acquisition**

Fluorescence images were captured using a Zeiss LSM 910 confocal laser scanning microscope (Carl Zeiss Microscopy AG, Oberkochen, Germany) equipped with 4D lightfield capabilities.

**Supplementary Figure S1**

**Supplementary Figure 1**. Detailed immunocytochemical analysis of HiBiT-tagged TAS2R42, TAS2R45, and TAS2R19 surface and total expression in HEK293T-Gα16gust44 cells. Cells were transiently transfected with HiBiT-tagged TAS2R expression constructs and incubated for ~24 h. To assess plasma membrane localization, live-cell immunostaining was performed by incubating cells with anti-HiBiT monoclonal antibody prior to fixation. Following washing and methanol/acetone fixation, HiBiT-tagged receptors were visualized using anti-mouse Alexa Fluor 488 secondary antibody (green). The plasma membrane was delineated by staining with biotinylated concanavalin A (prior to fixation) followed by streptavidin-Alexa Fluor 633 (red), and nuclei were counterstained with DAPI (blue). To evaluate total (surface plus intracellular) receptor distribution, a permeabilization protocol was employed: cells were first labeled with concanavalin A, then fixed, and subsequently incubated with anti-HiBiT antibody to allow access to intracellular epitopes. All samples were imaged using a Zeiss LSM 910 confocal laser-scanning microscope. Merged images display overlapping green (TAS2R-HiBiT), red (concanavalin A), and blue (DAPI) channels. A scale bar is provided in the lower right corner of the images. The figure compares surface-restricted versus permeabilized staining patterns for TAS2R42M (A, B, E, F, G) and TAS2R45M (C, D, H, I, J), two receptors almost absent from cell surfaces, and, as a control, the TAS2R19M (K-N) showing robust cell-surface expression. The insets with the corresponding line scanning graphs show more detailed the cell surface and intracellular localization of the chosen constructs. The inset in the upper left corresponds to the single green cell visible in A, all other insets are grouped by gray boxes to the corresponding original images.

The experiment revealed distinct subcellular localization profiles among these receptors. Whereas HiBiT-antiserum treatment prior to permeabilizing methanol/acetone fixation resulted in hardly any green signals in TAS2R42M (A, E, and corresponding inset and line scan) and TAS2R45M (C, H,) transfected cells, TAS2R19M was detected in numerous cells (K, L). Moreover, TAS2R19M showed green signals restricted to the plasma membrane level (also indicated by the overlap of green and red signals at the cell borders of the inset and line scan). In contrast to this, with anti-HiBiT-staining after methanol/acetone-fixation also green signals were obtained for TAS2R42M (B, F, G) and TAS2R45M (D, I, J, and corresponding inset and line scan) transfected cells, suggesting that the corresponding proteins are produced but trapped in intracellular compartments. Under these conditions, TAS2R19M showed, in addition to cell-surface immunoreactivity, large vesicular aggregates at intracellular sites (M, N, and corresponding inset and line scan).

In summary, this experiment demonstrated the exclusive labeling of cell-surface-associated TAS2R proteins when the HiBiT-antiserum was applied onto living non-permeabilized cells, as well as the restricted expression of TAS2R42M and TAS2R45M at intracellular sites.

**Supplementary Table S1.**

Amino acid sequences of the used TAS2Rs and their variants (variant positions are underscored):

HiBiT-tag (red), sst3-tag (green), TAS2R coding region (magenta), HSV-tag (blue).

TAS2R9 (nontaster variant)

**MVSGWRLFKKISMAAVTYPSSVPTTLDPGNASSAWPLDTSLGNASAGTSLAGLAVSGEFMPSAIEAIYIILIAGELTIGIWGNGFIVLVNCIDWLKRRDISLIDIILISLAISRICLLCVISLDGFFMLLFPGTYGNSVLVSIVNVVWTFANNSSLWFTSCLSIFYLLKIANISHPFFFWLKLKINKVMLAILLGSFLISLIISVPKNDDMWYHLFKVSHEENITWKFKVSKIPGTFKQLTLNLGVMVPFILCLISFFLLLFSLVRHTKQIRLHATGFRDPSTEAHMRAIKAVIIFLLLLIVYYPVFLVMTSSALIPQGKLVLMIGDIVTVIFPSSHSFILIMGNSKLREAFLKMLRFVKCFLRRRKPFVPGGRQPELAPEDPED**

TAS2R9 (taster variant)

**MVSGWRLFKKISMAAVTYPSSVPTTLDPGNASSAWPLDTSLGNASAGTSLAGLAVSGEFMPSAIEAIYIILIAGELTIGIWGNGFIVLVNCIDWLKRRDISLIDIILISLAISRICLLCVISLDGFFMLLFPGTYGNSVLVSIVNVVWTFANNSSLWFTSCLSIFYLLKIANISHPFFFWLKLKINKVMLAILLGSFLISLIISVPKNDDMWYHLFKVSHEENITWKFKVSKIPGTFKQLTLNLGAMVPFILCLISFFLLLFSLVRHTKQIRLHATGFRDPSTEAHMRAIKAVIIFLLLLIVYYPVFLVMTSSALIPQGKLVLMIGDIVTVIFPSSHSFILIMGNSKLREAFLKMLRFVKCFLRRRKPFVPGGRQPELAPEDPED**

TAS2R38-AVI (nontaster variant)

**MVSGWRLFKKISMAAVTYPSSVPTTLDPGNASSAWPLDTSLGNASAGTSLAGLAVSGEFMLTLTRIRTVSYEVRSTFLFISVLEFAVGFLTNAFVFLVNFWDVVKRQALSNSDCVLLCLSISRLFLHGLLFLSAIQLTHFQKLSEPLNHSYQAIIMLWMIANQANLWLAACLSLLYCSKLIRFSHTFLICLASWVSRKISQMLLGIILCSCICTVLCVWCFFSRPHFTVTTVLFMNNNTRLNWQNKDLNLFYSFLFCYLWSVPPFLLFLVSSGMLTVSLGRHMRTMKVYTRNSRDPSLEAHIKALKSLVSFFCFFVISSCVAFISVPLLILWRDKIGVMVCVGIMAACPSGHAAILISGNAKLRRAVMTILLWAQSSLKVRADHKADSRTLCGGRQPELAPEDPED**

TAS2R38-PAV (taster variant)

**MVSGWRLFKKISMAAVTYPSSVPTTLDPGNASSAWPLDTSLGNASAGTSLAGLAVSGEFMLTLTRIRTVSYEVRSTFLFISVLEFAVGFLTNAFVFLVNFWDVVKRQPLSNSDCVLLCLSISRLFLHGLLFLSAIQLTHFQKLSEPLNHSYQAIIMLWMIANQANLWLAACLSLLYCSKLIRFSHTFLICLASWVSRKISQMLLGIILCSCICTVLCVWCFFSRPHFTVTTVLFMNNNTRLNWQNKDLNLFYSFLFCYLWSVPPFLLFLVSSGMLTVSLGRHMRTMKVYTRNSRDPSLEAHIKALKSLVSFFCFFVISSCAAFISVPLLILWRDKIGVMVCVGIMAACPSGHAAVLISGNAKLRRAVMTILLWAQSSLKVRADHKADSRTLCGGRQPELAPEDPED**

TAS2R19

**MVSGWRLFKKISMAAVTYPSSVPTTLDPGNASSAWPLDTSLGNASAGTSLAGLAVSGEFMMCFLLIISSILVVFAFVLGNVANGFIALVNVIDWVNTRKISSAEQILTALVVSRIGLLWVMLFLWYATVFNSALYGLEVRIVASNAWAVTNHFSMWLAASLSIFCLLKIANFSNLISLHLKKRIKSVVLVILLGPLVFLICNLAVITMDERVWTKEYEGNVTWKIKLRNAIHLSSLTVTTLANLIPFTLSLICFLLLICSLCKHLKKMRLHSKGSQDPSTKVHIKALQTVTSFLMLFAIYFLCIITSTWNLRTQQSKLVLLLCQTVAIMYPSFHSFILIMGSRKLKQTFLSVLWQMTRGGRQPELAPEDPED**

TAS2R42

**MVSGWRLFKKISMAAVTYPSSVPTTLDPGNASSAWPLDTSLGNASAGTSLAGLAVSGEFMATELDKIFLILAIAEFIISMLGNVFIGLVNCSEGIKNQKVFSADFILTCLAISTIGQLLVILFDSFLVGLASHLYTTYRLGKTVIMLWHMTNHLTTWLATCLSIFYFFKIAHFPHSLFLWLRWRMNGMIVMLLILSLFLLIFDSLVLEIFIDISLNIIDKSNLTLYLDESKTLFDKLSILKTLLSLTSFIPFSLSLTSLLFLFLSLVRHTRNLKLSSLGSRDSSTEAHRRAMKMVMSFLFLFIVHFFSLQVANWIFFMLWNNKYIKFVMLALNAFPSCHSFILILGNSKLRQTAVRLLWHLRNYTKTPNALPLGGRQPELAPEDPED**

TAS2R45

**MVSGWRLFKKISMAAVTYPSSVPTTLDPGNASSAWPLDTSLGNASAGTSLAGLAVSGEFMITFLPIIFSILVVVTFVIGNFANGFIALVNSTEWVKRQKISFADQIVTALAVSRVGLLWVLLLNWYSTVLNPAFYSVELRTTAYNIWAVTGHFSNWPATSLSIFYLLKIANFSNLIFLRLKRRVKSVILVVLLGPLLFLACHLFVVNMNQIVWTKEYEGNMTWKIKLRRAMYLSDTTVTMLANLVPFTVTLISFLLLVCSLCKHLKKMQLHGKGSQDPSTKVHIKVLQTVISFFLLRAIYFVSVIISVWSFKNLENKPVFMFCQAIGFSCSSAHPFILIWGNKKLKQTYLSVLWQMRYGGRQPELAPEDPED**

TAS2R60

**MVSGWRLFKKISMAAVTYPSSVPTTLDPGNASSAWPLDTSLGNASAGTSLAGLAVSGEFMNGDHMVLGSSVTDKKAIILVTILLLLRLVAIAGNGFITAALGVEWVLRRMLLPCDKLLVSLGASRFCLQSVVMGKTIYVFLHPMAFPYNPVLQFLAFQWDFLNAATLWSSTWLSVFYCVKIATFTHPVFFWLKHKLSGWLPWMLFSSVGLSSFTTILFFIGNHRMYQNYLRNHLQPWNVTGDSIRSYCEKFYLFPLKMITWTMPTAVFFICMILLITSLGRHRKKALLTTSGFREPSVQAHIKALLALLSFAMLFISYFLSLVFSAAGIFPPLDFKFWVWESVIYLCAAVHPIILLFSNCRLRAVLKSRRSSRCGTPGGRQPELAPEDPED**

**Supplementary Table S2.** Detailed statistics for luminometric measurements shown in Figures 2, 3, and 4 of the main manuscript. Statistics were calculated using Graph pad prism ANOVA software with the indicated post hoc tests.

| The non-taster variants of TAS2R9 and TAS2R38 are significantly less expressed at the cell surface as the corresponding taster variants (Figure 2D main manuscript) | | | |
| --- | --- | --- | --- |
| Tukey's multiple comparisons test | Mean Diff. | 95.00% CI of diff. | Adjusted P Value |
| TAS2R9M vs. TAS2R9NTM | 9.583 | 6.313 to 12.85 | **<0.0001** |
| TAS2R38 vs. TAS2R38NTM | 53.18 | 43.96 to 62.40 | **<0.0001** |
|  |  |  |  |
| The orphan receptors TAS2R42 and TAS2R45 are significantly expressed at the cell surface, when compared to HiBiT-tag-free TAS2R45 (Figure 3A main manuscript) | | | |
| Dunnett's multiple comparisons test | Mean Diff. | 95.00% CI of diff. | Adjusted P Value |
| TAS2R45 vs. TAS2R45M | -1.218 | -1.290 to -1.145 | **<0.0001** |
| TAS2R45 vs. TAS2R42M | -20.62 | -25.99 to -15.24 | **0.0001** |
|  |  |  |  |
| Compared to a negative control (HiBiT-free TAS2R14) TAS2R14M, TAS2R19M, and TAS2R60M are significantly detected at the cell surface (Figure 3B main manuscript) | | | |
| Dunnett's multiple comparisons test | Mean Diff. | 95.00% CI of diff. | Adjusted P Value |
| TAS2R14 vs. TAS2R19M | -511.2 | -562.5 to -459.9 | **<0.0001** |
| TAS2R14 vs. TAS2R60M | -226.8 | -278.2 to -175.5 | **<0.0001** |
| TAS2R14 vs. TAS2R14M | -202.6 | -255.6 to -149.6 | **<0.0001** |

| Detailed statistical evaluation of the data shown in Figure 4 | | | |
| --- | --- | --- | --- |
| Tukey's multiple comparisons test | Mean Diff. | 95.00% CI of diff. | Adjusted P Value |
| TAS2R9M vs. TAS2R9NTM | -22.3 | -199.0 to 154.4 | >0.9999 |
| TAS2R9M vs. TAS2R38-PAVM | -257.9 | -434.6 to -81.19 | **0.0003** |
| TAS2R9M vs. TAS2R38-AVIM | -115 | -291.7 to 61.74 | 0.5299 |
| TAS2R9M vs. TAS2R42M | -235.7 | -412.4 to -58.98 | **0.0014** |
| TAS2R9M vs. TAS2R45M | 29.6 | -161.3 to 220.5 | >0.9999 |
| TAS2R9M vs. TAS2R19M | -412.6 | -589.3 to -235.9 | **<0.0001** |
| TAS2R9M vs. TAS2R60M | -1643 | -1819 to -1466 | **<0.0001** |
| TAS2R9M vs. TAS2R14M | -600.9 | -777.7 to -424.2 | **<0.0001** |
| TAS2R9M vs. TAS2R14w/o HiBiT | 59.85 | -116.9 to 236.6 | 0.9843 |
| TAS2R9NTM vs. TAS2R38-PAVM | -235.6 | -412.3 to -58.89 | **0.0015** |
| TAS2R9NTM vs. TAS2R38-AVIM | -92.68 | -269.4 to 84.04 | 0.796 |
| TAS2R9NTM vs. TAS2R42M | -213.4 | -390.1 to -36.68 | **0.0062** |
| TAS2R9NTM vs. TAS2R45M | 51.89 | -139.0 to 242.8 | 0.9968 |
| TAS2R9NTM vs. TAS2R19M | -390.3 | -567.0 to -213.6 | **<0.0001** |
| TAS2R9NTM vs. TAS2R60M | -1620 | -1797 to -1444 | **<0.0001** |
| TAS2R9NTM vs. TAS2R14M | -578.6 | -755.4 to -401.9 | **<0.0001** |
| TAS2R9NTM vs. TAS2R14w/o HiBiT | 82.15 | -94.58 to 258.9 | 0.8885 |
| TAS2R38-PAVM vs. TAS2R38-AVIM | 142.9 | -33.79 to 319.7 | 0.2243 |
| TAS2R38-PAVM vs. TAS2R42M | 22.21 | -154.5 to 198.9 | >0.9999 |
| TAS2R38-PAVM vs. TAS2R45M | 287.5 | 96.63 to 478.4 | **0.0002** |
| TAS2R38-PAVM vs. TAS2R19M | -154.7 | -331.4 to 22.06 | 0.1404 |
| TAS2R38-PAVM vs. TAS2R60M | -1385 | -1562 to -1208 | **<0.0001** |
| TAS2R38-PAVM vs. TAS2R14M | -343 | -519.7 to -166.3 | **<0.0001** |
| TAS2R38-PAVM vs. TAS2R14w/o HiBiT | 317.8 | 141.0 to 494.5 | **<0.0001** |
| TAS2R38-AVIM vs. TAS2R42M | -120.7 | -297.4 to 56.00 | 0.4583 |
| TAS2R38-AVIM vs. TAS2R45M | 144.6 | -46.31 to 335.5 | 0.3091 |
| TAS2R38-AVIM vs. TAS2R19M | -297.6 | -474.3 to -120.9 | **<0.0001** |
| TAS2R38-AVIM vs. TAS2R60M | -1528 | -1704 to -1351 | **<0.0001** |
| TAS2R38-AVIM vs. TAS2R14M | -486 | -662.7 to -309.2 | **<0.0001** |
| TAS2R38-AVIM vs. TAS2R14w/o HiBiT | 174.8 | -1.897 to 351.5 | 0.055 |
| TAS2R42M vs. TAS2R45M | 265.3 | 74.41 to 456.2 | **0.0007** |
| TAS2R42M vs. TAS2R19M | -176.9 | -353.6 to -0.1559 | **0.0496** |
| TAS2R42M vs. TAS2R60M | -1407 | -1584 to -1230 | **<0.0001** |
| TAS2R42M vs. TAS2R14M | -365.2 | -542.0 to -188.5 | **<0.0001** |
| TAS2R42M vs. TAS2R14w/o HiBiT | 295.5 | 118.8 to 472.3 | **<0.0001** |
| TAS2R45M vs. TAS2R19M | -442.2 | -633.1 to -251.3 | **<0.0001** |
| TAS2R45M vs. TAS2R60M | -1672 | -1863 to -1481 | **<0.0001** |
| TAS2R45M vs. TAS2R14M | -630.5 | -821.4 to -439.7 | **<0.0001** |
| TAS2R45M vs. TAS2R14w/o HiBiT | 30.25 | -160.6 to 221.1 | >0.9999 |
| TAS2R19M vs. TAS2R60M | -1230 | -1407 to -1053 | **<0.0001** |
| TAS2R19M vs. TAS2R14M | -188.4 | -365.1 to -11.64 | **0.027** |
| TAS2R19M vs. TAS2R14w/o HiBiT | 472.4 | 295.7 to 649.1 | **<0.0001** |
| TAS2R60M vs. TAS2R14M | 1042 | 865.0 to 1218 | **<0.0001** |
| TAS2R60M vs. TAS2R14w/o HiBiT | 1703 | 1526 to 1879 | **<0.0001** |
| TAS2R14M vs. TAS2R14w/o HiBiT | 660.8 | 484.1 to 837.5 | **<0.0001** |
|  |  |  |  |
| The cell surface fractions of the employed TAS2Rs deviates significantly | | | |
| Tukey's multiple comparisons test | Mean Diff. | 95.00% CI of diff. | Adjusted P Value |
| TAS2R9M vs. TAS2R9NTM | 0.2284 | 0.1224 to 0.3344 | **<0.0001** |
| TAS2R9M vs. TAS2R38-PAVM | 0.1771 | 0.07105 to 0.2831 | **<0.0001** |
| TAS2R9M vs. TAS2R38-AVIM | 0.2749 | 0.1689 to 0.3809 | **<0.0001** |
| TAS2R9M vs. TAS2R42M | 0.3524 | 0.2464 to 0.4584 | **<0.0001** |
| TAS2R9M vs. TAS2R45M | 0.3704 | 0.2028 to 0.5380 | **<0.0001** |
| TAS2R9M vs. TAS2R19M | -0.6518 | -0.7579 to -0.5458 | **<0.0001** |
| TAS2R9M vs. TAS2R60M | 0.2947 | 0.1887 to 0.4007 | **<0.0001** |
| TAS2R9M vs. TAS2R14M | 0.1213 | 0.01153 to 0.2310 | **0.0177** |
| TAS2R9M vs. TAS2R14w/o HiBiT | -0.09916 | -0.2089 to 0.01058 | 0.115 |
| TAS2R9NTM vs. TAS2R38-PAVM | -0.05136 | -0.1574 to 0.05466 | 0.8702 |
| TAS2R9NTM vs. TAS2R38-AVIM | 0.04649 | -0.05953 to 0.1525 | 0.9253 |
| TAS2R9NTM vs. TAS2R42M | 0.124 | 0.01798 to 0.2300 | **0.0088** |
| TAS2R9NTM vs. TAS2R45M | 0.142 | -0.02565 to 0.3096 | 0.1769 |
| TAS2R9NTM vs. TAS2R19M | -0.8803 | -0.9863 to -0.7742 | **<0.0001** |
| TAS2R9NTM vs. TAS2R60M | 0.06631 | -0.03971 to 0.1723 | 0.5996 |
| TAS2R9NTM vs. TAS2R14M | -0.1072 | -0.2169 to 0.002584 | 0.062 |
| TAS2R9NTM vs. TAS2R14w/o HiBiT | -0.3276 | -0.4373 to -0.2178 | **<0.0001** |
| TAS2R38-PAVM vs. TAS2R38-AVIM | 0.09785 | -0.008174 to 0.2039 | 0.0983 |
| TAS2R38-PAVM vs. TAS2R42M | 0.1754 | 0.06934 to 0.2814 | **<0.0001** |
| TAS2R38-PAVM vs. TAS2R45M | 0.1933 | 0.02571 to 0.3610 | **0.0105** |
| TAS2R38-PAVM vs. TAS2R19M | -0.8289 | -0.9349 to -0.7229 | **<0.0001** |
| TAS2R38-PAVM vs. TAS2R60M | 0.1177 | 0.01164 to 0.2237 | **0.0168** |
| TAS2R38-PAVM vs. TAS2R14M | -0.0558 | -0.1655 to 0.05394 | 0.8337 |
| TAS2R38-PAVM vs. TAS2R14w/o HiBiT | -0.2762 | -0.3860 to -0.1665 | **<0.0001** |
| TAS2R38-AVIM vs. TAS2R42M | 0.07751 | -0.02851 to 0.1835 | 0.3689 |
| TAS2R38-AVIM vs. TAS2R45M | 0.09549 | -0.07214 to 0.2631 | 0.7205 |
| TAS2R38-AVIM vs. TAS2R19M | -0.9267 | -1.033 to -0.8207 | **<0.0001** |
| TAS2R38-AVIM vs. TAS2R60M | 0.01982 | -0.08620 to 0.1258 | 0.9999 |
| TAS2R38-AVIM vs. TAS2R14M | -0.1536 | -0.2634 to -0.04391 | **0.0005** |
| TAS2R38-AVIM vs. TAS2R14w/o HiBiT | -0.3741 | -0.4838 to -0.2643 | **<0.0001** |
| TAS2R42M vs. TAS2R45M | 0.01798 | -0.1497 to 0.1856 | >0.9999 |
| TAS2R42M vs. TAS2R19M | -1.004 | -1.110 to -0.8982 | **<0.0001** |
| TAS2R42M vs. TAS2R60M | -0.0577 | -0.1637 to 0.04832 | 0.7708 |
| TAS2R42M vs. TAS2R14M | -0.2312 | -0.3409 to -0.1214 | **<0.0001** |
| TAS2R42M vs. TAS2R14w/o HiBiT | -0.4516 | -0.5613 to -0.3418 | **<0.0001** |
| TAS2R45M vs. TAS2R19M | -1.022 | -1.190 to -0.8546 | **<0.0001** |
| TAS2R45M vs. TAS2R60M | -0.07568 | -0.2433 to 0.09196 | 0.9117 |
| TAS2R45M vs. TAS2R14M | -0.2491 | -0.4191 to -0.07913 | **0.0002** |
| TAS2R45M vs. TAS2R14w/o HiBiT | -0.4696 | -0.6396 to -0.2996 | **<0.0001** |
| TAS2R19M vs. TAS2R60M | 0.9466 | 0.8405 to 1.053 | **<0.0001** |
| TAS2R19M vs. TAS2R14M | 0.7731 | 0.6634 to 0.8828 | **<0.0001** |
| TAS2R19M vs. TAS2R14w/o HiBiT | 0.5527 | 0.4429 to 0.6624 | **<0.0001** |
| TAS2R60M vs. TAS2R14M | -0.1735 | -0.2832 to -0.06372 | **<0.0001** |
| TAS2R60M vs. TAS2R14w/o HiBiT | -0.3939 | -0.5036 to -0.2841 | **<0.0001** |
| TAS2R14M vs. TAS2R14w/o HiBiT | -0.2204 | -0.3338 to -0.1071 | **<0.0001** |

**Supplementary Table S3**. Raw data of cell surface and total luminescence detection (RLU = relative light units). Average values (Mean), standard error of the mean (SEM), and number of measurements (N) are provided.

|  | **Construct (TAS2R...)** | | | | | | | | | | |
| --- | --- | --- | --- | --- | --- | --- | --- | --- | --- | --- | --- |
|  | **9M** | **9NTM** | **38-PAVM** | **38-AVIM** | **42M** | **45M** | **19M** | **60M** | **14M** | **14w/o HiBiT** | **45w/o HiBiT** |
| Cell surface  RLU | 23 | 16 | 72 | 26 | 29 | 2 | 443 | 164 | 242 | 1 | 1 |
|  | 18 | 15 | 82 | 26 | 15 | 2 | 509 | 184 | 212 | 1 | 0 |
|  | 19 | 13 | 50 | 23 | 13 | 2 | 432 | 179 | 161 | 1 | 1 |
|  | 26 | 16 | 56 | 27 | 18 | 2 | 502 | 143 | 170 | 1 | 1 |
|  | 26 | 13 | 58 | 15 | 22 | 2 | 411 | 132 | 192 | 2 | 1 |
|  | 30 | 16 | 88 | 22 | 19 | 2 | 504 | 156 | 240 | 1 | 1 |
|  | 26 | 12 | 67 | 15 | 20 |  | 504 | 215 | 230 | 1 |  |
|  | 28 | 15 | 85 | 24 | 23 |  | 522 | 233 | 160 | 0 |  |
|  | 30 | 21 | 105 | 24 | 20 |  | 463 | 293 | 116 | 1 |  |
|  | 25 | 18 | 95 | 36 | 23 |  | 538 | 318 | 212 | 1 |  |
|  | 26 | 17 | 64 | 25 | 8 |  | 256 | 218 | 114 | 1 |  |
|  | 35 | 15 | 87 | 22 | 21 |  | 450 | 278 | 163 | 1 |  |
|  | 36 | 15 | 82 | 24 | 16 |  | 388 | 254 | 293 | 1 |  |
|  | 27 | 13 | 54 | 18 | 8 |  | 539 | 264 | 145 | 1 |  |
|  | 28 | 20 | 85 | 20 | 20 |  | 450 | 326 | 236 | 1 |  |
|  | 29 | 15 | 103 | 24 | 17 |  | 410 | 284 | 98 | 1 |  |
|  | 27 | 22 | 122 | 39 | 43 |  | 587 | 273 | 245 | 1 |  |
|  | 19 | 16 | 110 | 41 | 33 |  | 728 | 224 | 255 | 1 |  |
|  | 17 | 20 | 85 | 37 | 29 |  | 508 | 215 | 255 | 1 |  |
|  | 27 | 19 | 90 | 35 | 32 |  | 567 | 175 | 236 | 1 |  |
|  | 20 | 21 | 74 | 30 | 27 |  | 473 | 213 | 295 | 1 |  |
|  | 26 | 17 | 60 | 30 | 31 |  | 811 | 240 |  |  |  |
|  | 34 | 16 | 64 | 32 | 29 |  | 596 | 270 |  |  |  |
|  | 29 | 17 | 88 | 36 | 27 |  | 696 | 215 |  |  |  |
| Mean | **26.3** | **16.7** | **80.3** | **27.1** | **22.6** | **1.8** | **512.1** | **227.7** | **203.4** | **0.9** | **0.7** |
| SEM | **1.01** | **0.54** | **3.87** | **1.50** | **1.67** | **0.08** | **23.76** | **11.05** | **12.55** | **0.07** | **0.09** |
| N | **24** | **24** | **24** | **24** | **24** | **6** | **24** | **24** | **21** | **21** | **6** |
|  |  |  |  |  |  |  |  |  |  |  |  |
| Total RLU | 71 | 91 | 511 | 202 | 414 | 35 | 690 | 1916 | 702 | 1 |  |
|  | 71 | 70 | 433 | 211 | 389 | 28 | 544 | 1306 | 809 | 2 |  |
|  | 52 | 87 | 295 | 239 | 393 | 31 | 479 | 1449 | 609 | 2 |  |
|  | 76 | 87 | 473 | 239 | 231 |  | 497 | 1795 | 786 | 1 |  |
|  | 65 | 90 | 279 | 194 | 289 | 42 | 410 | 2149 | 760 | 1 |  |
|  | 52 | 76 | 217 | 160 | 243 | 34 | 500 | 2067 | 687 | 2 |  |
|  | 51 | 84 | 237 | 187 | 298 | 35 | 464 | 2147 | 727 | 2 |  |
|  | 56 | 89 | 330 | 142 | 259 |  | 464 | 2134 | 721 | 1 |  |
|  | 68 | 76 | 262 | 146 | 341 | 27 | 492 | 1564 | 639 | 1 |  |
|  | 61 | 82 | 252 | 125 | 232 | 27 | 434 | 1173 | 555 | 2 |  |
|  | 53 | 96 | 262 | 130 | 279 | 29 | 382 | 1405 | 444 | 1 |  |
|  | 61 | 79 | 283 | 142 | 199 |  | 332 | 1345 | 510 | 2 |  |
| Mean | **61.5** | **83.8** | **319.4** | **176.5** | **297.2** | **31.9** | **474.0** | **1704.2** | **662.4** | **1.6** |  |
| SEM | **2.51** | **2.16** | **28.24** | **11.87** | **20.64** | **1.63** | **25.79** | **106.91** | **32.76** | **0.12** |  |
| N | **12** | **12** | **12** | **12** | **12** | **9** | **12** | **12** | **12** | **12** |  |

**Supplementary Table S4.** Replicate level numerical data for experiments.

| Figure No. | Number of experiments |
| --- | --- |
| 2A | N=4 (2 wells in 2 exp. and 3 wells in 2 exp.) |
| 2B | N=5 (2 wells in 3 exp. and 3 wells in 2 exp.) |
| 2C | Sum of N=3 (8 wells each) |
| 2D | N=3 (8 wells each) |
| 3 | N=3 (8 wells each; except TAS2R45 2 wells each) |
| 4 | N=3 (3 wells each) |
| 5 anti HiBiT | N=1 (2 coverslips each; TAS2R9, -R9NT, -R38, -R38NT, -R19, -R42, -R45, -R60) |
| 6 anti HSV | N=2 (2 coverslips each; TAS2R9, -R9NT), N=1 (2 coverslips each; TAS2R38, -R38NT, -R19, -R42, -R45, -R60, -R14) |
| S1 anti HiBiT | N=2 (2 coverslips each; w/o permeabilization), N=1 (2 coverslips each; with permeabilization) |
